# Mitotic Errors Promote Genomic Instability and Leukemia in a Novel Mouse Model of Fanconi Anemia

**DOI:** 10.3389/fonc.2021.752933

**Published:** 2021-11-05

**Authors:** Donna M. Edwards, Dana K. Mitchell, Zahi Abdul-Sater, Ka-Kui Chan, Zejin Sun, Aditya Sheth, Ying He, Li Jiang, Jin Yuan, Richa Sharma, Magdalena Czader, Pei-Ju Chin, Yie Liu, Guillermo de Cárcer, Grzegorz Nalepa, Hal E. Broxmeyer, D. Wade Clapp, Elizabeth A. Sierra Potchanant

**Affiliations:** ^1^ Department of Pediatrics, Division of Pediatric Hematology-Oncology, Indiana University School of Medicine, Indianapolis, IN, United States; ^2^ Department of Biochemistry and Molecular Biology, Indiana University School of Medicine, Indianapolis, IN, United States; ^3^ Wells Center for Pediatric Research, Indiana University School of Medicine, Indianapolis, IN, United States; ^4^ Department of Medical and Molecular Genetics, Indiana University School of Medicine, Indianapolis, IN, United States; ^5^ Department of Pediatrics, Riley Hospital for Children, Indianapolis, IN, United States; ^6^ Department of Pathology and Laboratory Medicine, Indiana University School of Medicine, Indianapolis, IN, United States; ^7^ Laboratory of Molecular Gerontology, Biomedical Research Center, National Institute on Aging, National Institutes of Health, Baltimore, MD, United States; ^8^ Cancer Biology Department, Instituto de Investigaciones Biomédicas “Alberto Sols” (IIBM), Consejo Superior de Investigaciones Científicas (CSIC), Madrid, Spain

**Keywords:** Fanconi anemia, leukemia, spindle assembly checkpoint, genomic instability, FANCC

## Abstract

Fanconi anemia (FA) is a disease of genomic instability and cancer. In addition to DNA damage repair, FA pathway proteins are now known to be critical for maintaining faithful chromosome segregation during mitosis. While impaired DNA damage repair has been studied extensively in FA-associated carcinogenesis *in vivo*, the oncogenic contribution of mitotic abnormalities secondary to FA pathway deficiency remains incompletely understood. To examine the role of mitotic dysregulation in FA pathway deficient malignancies, we genetically exacerbated the baseline mitotic defect in *Fancc-/-* mice by introducing heterozygosity of the key spindle assembly checkpoint regulator *Mad2*. *Fancc-/-;Mad2+/-* mice were viable, but died from acute myeloid leukemia (AML), thus recapitulating the high risk of myeloid malignancies in FA patients better than *Fancc-/-*mice. We utilized hematopoietic stem cell transplantation to propagate *Fancc-/-; Mad2+/-* AML in irradiated healthy mice to model *FANCC*-deficient AMLs arising in the non-FA population. Compared to cells from *Fancc-/-* mice, those from *Fancc-/-;Mad2+/-* mice demonstrated an increase in mitotic errors but equivalent DNA cross-linker hypersensitivity, indicating that the cancer phenotype of *Fancc-/-;Mad2+/-* mice results from error-prone cell division and not exacerbation of the DNA damage repair defect. We found that FANCC enhances targeting of endogenous MAD2 to prometaphase kinetochores, suggesting a mechanism for how FANCC-dependent regulation of the spindle assembly checkpoint prevents chromosome mis-segregation. Whole-exome sequencing revealed similarities between human FA-associated myelodysplastic syndrome (MDS)/AML and the AML that developed in *Fancc-/-; Mad2+/-* mice. Together, these data illuminate the role of mitotic dysregulation in FA-pathway deficient malignancies *in vivo*, show how FANCC adjusts the spindle assembly checkpoint rheostat by regulating MAD2 kinetochore targeting in cell cycle-dependent manner, and establish two new mouse models for preclinical studies of AML.

## Introduction

Fanconi anemia (FA) is a heterogeneous genetic disorder characterized by bone marrow failure, developmental abnormalities, and cancer predisposition (1). Germline mutations in any one of the 22 genes that encode FA pathway proteins causes Fanconi Anemia, whereas monoallelic inactivation of specific FA genes, such as *BRCA1* and *BRCA2*, results in an adult-onset predisposition for breast, ovarian, and pancreatic cancer (1). Furthermore, inactivation of FA pathway proteins *via* somatic mutation or epigenetic silencing is now being recognized as a key event contributing to the development of several types of sporadic cancers, including more than 40% of acute myelogenous leukemias (AML) (1–3).

The increased risk of malignant transformation in the setting of FA-deficiency is ultimately a consequence of increased genomic instability. However, the specific mechanisms underlying the origins of this genomic instability are incompletely understood (4). The best characterized role of the FA pathway is in the detection and repair of DNA damage (1). Although several mouse models have been developed to study the molecular pathogenesis of FA, most have been unable to precisely recapitulate the hematologic malignancies of human FA patients [reviewed in (5)]. *Fancc*-/- mice demonstrate many of the phenotypic characteristics consistent with FA pathway deficiency, including hypersensitivity to DNA cross-linking agents and impaired hematopoietic stem cell repopulating capacity (6, 7). However, our group previously demonstrated that while aged *Fancc*-/- mice eventually develop hematologic malignancies consistent with human AML, they do not develop early onset disease like their human FA counterparts. Importantly, we found that leukemogenesis in *Fancc*-/- mice was preceded by an increase in abnormal mitotic figures and aneuploidy, which was noted to be exacerbated upon malignant transformation (8). Consistent with these observations, we have previously shown that loss of FA pathway proteins results in a weakening of the spindle assembly checkpoint (SAC), a complex signaling cascade that delays anaphase onset until each sister chromatid is properly attached to the mitotic spindle (4, 9). These findings suggest that defects in both DNA damage repair and mitotic fidelity may contribute to progressive genomic instability in the setting of FA pathway compromise.

Accordingly, emerging evidence suggests that impaired DNA damage repair and compromised mitotic fidelity may cooperate to facilitate tumorigenesis (10). Specifically, previous studies have suggested that FA pathway disruption and SAC inactivation cooperate to promote malignant transformation. In their elegant study, Lee et al., demonstrated that inactivation of the SAC through loss of BUB1 function promoted transformation in cells lacking BRCA2, a prevalent FA pathway protein (1, 11–13). Additionally, patients with FA pathway mutations are thought to be at an increased risk of developing HPV-associated cancers (14). Interestingly, a recent study demonstrated that the HPV E6 protein disrupts the SAC by causing a decrease in kinetochore-associated MAD2 (15). Given these results and our previous findings, we hypothesized that FA-deficient cells are hyper-dependent on the SAC and that further SAC compromise would be sufficient to drive leukemogenesis in the setting of FA pathway dysfunction.

To test this hypothesis, we generated a novel mouse model in which the weakened SAC of *Fancc-/-* mice was further impaired by direct genetic disruption. For this model, it was necessary to select a component of the SAC that could be manipulated without altering the gross structure of the kinetochore or other important mitotic pathways. Previous studies have suggested a role for the SAC protein BUB1B in promoting leukemogenesis (16, 17). However, in addition to its role in the SAC, BUB1B is also known to be critical for chromosome alignment, end-on capture of microtubules, and chromosome congression (18–20). Likewise, the SAC protein BUB1 is required for kinetochore localization of BUB1B, centromere proteins CENP-E and CENP-F, as well as for proper chromosome congression and sister centromere cohesion (21, 22). Lastly, the recruitment of BUB1 and BUB1B to the kinetochore is dependent upon BUB3 (23). Therefore, given their important SAC-independent mitotic functions, we excluded *Bub1*, *Bub1b*, and *Bub3* from consideration for our model (24, 25). Complete loss of the SAC component *Mad2* results in embryonic lethality, but *Mad2* heterozygous mice develop normally and are not known to develop cancer at a young age (26). Additionally, *Mad2+/-* mice do not develop hematologic malignancies and develop solid tumors only after a long latency period, suggesting that additional events are required to drive tumorigenesis (26). Thus, we reasoned that introducing *Mad2* heterozygosity into *Fancc-/-* mice would allow for the specific evaluation of the contribution of SAC insufficiency to leukemogenesis in the setting of *Fancc*-deficiency (6, 27).

In this study, we strategically crossed *Fancc-/-* and *Mad+/-* mice to further genetically compromise the SAC. As we hypothesized, heterozygous deletion of *Mad2* accelerated the onset of hematologic malignancies in *Fancc-/-* mice, resulting predominantly in AML. Compared to other FA murine models, this phenotype more closely recapitulates the childhood AML predisposition of human FA patients (1). Importantly, the increased incidence of hematologic malignant transformation was associated with an exacerbation of the underlying SAC impairment, but not of the underlying DNA damage repair defect. This novel AML-prone mouse model provides a valuable tool for studying leukemogenesis in FA and provides *in vivo* evidence implicating SAC insufficiency as a driver of tumorigenesis in the setting of FA pathway deficiency.

## Methods

### Mice

*Mad2+/-* and *Fancc-/-* mice (C57Bl/6J background) were a gift of D. Wade Clapp (Indiana University).

Mice were genotyped as described (28). B6.SJL-Ptprc^a^Pepc^b^/BoyJ mice were purchased from IU *In Vivo* Therapeutics Core. All studies were performed in accordance with the policies of the IU IACUC.

### Peripheral Blood Assays

Blood was drawn from lateral tail veins and collected into EDTA-coated tubes. Complete blood counts were obtained using a Hemavet 950FS (Drew Scientific). Blood smears were stained with Wright-Giemsa. For competitive transplant experiments, cells were stained with anti-CD45.2-FITC and anti-CD45.1-PE (BD Biosciences) as described (28) and analyzed on FacsCalibur (Becton-Dickinson). For lineage WBC classification, the anti-CD3e (BD Bioscience), anti-B220 (clone RA3-6B2, BD Pharmingen), anti-Cd11b (clone M1/70, eBioscience), and anti-Ly-6G/Ly-6C (clone RB6-8C5, Biolegend) were used. At least 10,000 events/sample were acquired. Data were analyzed using FlowJo.

### Bone Marrow Harvest/Transplantation

Bone marrow cells were flushed from tibias/femurs of mice using a 23-gauge needle (Becton Dickinson). LDMNCs were isolated by density gradient using Histopaque-1119 (Sigma), centrifuging for 30 minutes (1800 rpm, no brake); LDMNCs were removed from the interface. Murine cytospins were made by resuspending LDMNCs in PBS and centrifuging onto slides (450rpm, 5 minutes) on a Shandon Cytospin-3 Cytocentrifuge (Thermo). For competitive transplant studies, 0.5-1 million donor test LDMNCs from *wt* or *Fancc-/-; Mad2+/-* (C57Bl/6J, CD45.2+) mice were transplanted with *wt* donor competitor LDMNCs (BoyJ, CD45.1+) *via* tail-vein injection into eight-week old female B6.SJL-Ptprc^a^Pepc^b^/BoyJ mice that underwent whole-body split-dose 1100 rads irradiation (700 rads/400 rads, 4 hours apart). A competitive transplant design was used to ensure recipient survival to evaluate engraftment and propagation of malignancy post-radiation.

### Histology

Mouse tissues were fixed in 10% formalin, paraffin-embedded, sectioned (5μM sections), and stained with hematoxylin and eosin (H&E). The following antibodies were used: anti-c-kit (C19, Santa Cruz, 1:50), anti-cd11b (Novus, 1:50), CD3 (Dako, IR503), B220 (Clone RA3-6B2, BD Pharmingen). Peripheral blood smears and marrow cytospins were stained with Giemsa using the Siemens Hematek 3000 system. Diagnoses of leukemia and lymphoma in the *Fancc-/-; Mad2+/-* mice were made using Bethesda criteria for the non-lymphoid and lymphoid neoplasms in mice (29) and confirmed by a mouse veterinary pathologist at IU School of Medicine. Confirmation of leukemia post-transplant was determined by primarily by histology upon presence of leukemic infiltrates in non-hematopoietic organs (such as liver, kidneys, lungs) at time of necropsy, as well as presence of blasts in the peripheral blood, marrow and/or spleen.

### Hematopoietic Progenitor Assays

20,000 LDMNCs were plated in triplicate onto 35x10mm dishes with 2mm grids (Thermo) and grown in methylcellulose (H4100 STEMCELL) media in IMDM containing 100 ng/ml m-SCF, 10 ng/ml mGM-CSF, 5 ng/ml mIL-3, 4 units/ml EPO, 2mM glutamine, 0.00005% βME, 30% FBS, 1% P/S and MMC at indicated concentrations. Cells were incubated at 37°C/5% CO_2_ for seven days and scored, with a colony defined as a cluster of ≥50 cells. Colonies were scored and reported as colony forming units per femur, accounting for donor marrow cellularity.

### Metaphase Spreads

Marrow cells were cultured in IMDM with 20% FBS, murine SCF (100ng/ml), and IL-6 (200ng/ml) for two days. Cells were then exposed to 0.2 μg/ml colcemid (Life Tech) for 4 hours and pelleted (800 rpm) for 5 minutes. Cells were resuspended dropwise in pre-warmed (37°C) 75mM KCl while vortexing gently and incubated at 37° C for 15 minutes. After pelleting, cells were resuspended in a 3:1 methanol: glacial acetic acid. Cells were pelleted and resuspended in fixative solution two additional times before being dropped onto slides and dried overnight. Spreads were then mounted in Vectashield medium with DAPI (Vector Laboratories). For the chromosome breakage test, cells were cultured for 48 hours in 50nM MMC before colcemid addition. For spectral karyotyping (SKY), samples were imaged and analyzed by the MD Anderson Cancer Center Molecular Cytogenetics Facility (Houston, TX).

### SAC Evaluation

C-kit+ cells were isolated from LDMNC population by MACs sorting (mouse CD117 beads, Miltenyi-Biotec), and cultured for 24 hours in 10% FBS, 1% P/S, 100ng/ml TPO, 100ng/ml SCF, and 100ng/ml Flt3 in RPMI. The cells were pulsed with 10uM EdU (2 hours) followed by 100 ng/ml nocodazole (12 hours). Cell fixation and EdU staining were performed using the Click-it EdU kit (Thermo) followed by phospho-H3/Alexa-647 staining (Cell Signaling). Cells were spun onto coverslips (450 rpm, 5min) and mounted with ProLong Diamond Antifade/DAPI (Thermo). EdU+ cells were counted on the deconvolution microscope (100x). BMDMs were obtained by culturing the LDMNCs in IMDM with 20% FBS, 10 ng/ml M-CSF, and 1% P/S for seven days.

### Microscopy

Histology images were obtained using a Zeiss Axiolab system. Metaphase images were acquired on a Deltavision personalDx deconvolution microscope (Applied Precision). Image stacks (distance between z-sections: 200 nM) were deconvolved using Softworx suite (10 iterations; ratio: conservative) and analyzed using Imaris (Bitplane). For live mitosis studies, a Biostation live-imaging microscope (Nikon) at 37°C/5% CO_2_ was used, with phase-contrast image stacks (z-sections distance: 2μm) captured in two-minute intervals for 24 hours. Videos were analyzed using NIS-Elements Viewer (Nikon).

### Immunofluorescence for MAD2-KT Quantification

MEFs were grown under the above indicated conditions and fixed on glass coverslips (Fisher Scientific) in 4% paraformaldehyde/0.5% Triton X-100 in PBS for 10 minutes at room temperature. Coverslips were washed with PBS-T (0.03% Triton X-100), and then permeabilized with 0.5% Triton X-100/0.05% SDS in PBS for 30 minutes at room temperature. The coverslips were then blocked in 10% FBS/0.03% Triton X-100 in PBS for 1 hour at room temperature. Coverslips were stained with rabbit polyclonal anti-human Mad2L1 (1:100 in PBS, Life Span Biosciences; LS-B13367) and mouse monoclonal anti-Hec1 (1:100 in PBS, Santa Cruz Biotechnology; SC-515550) for 2 hours at room temperature, then washed three times with PBST-T, 5 minutes per wash. Coverslips were then incubated with fluorophore-conjugated secondary antibodies (1:2000 in PBS, Life Technologies) at room temperature. Coverslips were washed three times in PBS-T, 5 minutes each, rinsed once in distilled water, then mounted in ProLong Diamond antifade mountant with DAPI (Molecular Probes) and cured at room temperature at least 24 hours. The fluorescence signals were captured using a Deltavision personalDx deconvolution microscope (Applied Precision). Image stacks (distance between z-sections: 200 nM) were deconvolved using Softworx suite (10 iterations; ratio: conservative). Quantification of fluorescence intensity at kinetochores and subsequent statistical analysis were performed using Imaris (Bitplane) and GraphPad Prism, respectively.

### Quantitative Western Blotting

Whole-cell lysates were prepared for Western blotting by incubating cells in M-PER mammalian protein extraction reagent (Life Technologies) with protease (Complete Mini, EDTA-free; Roche) and phosphatase inhibitors (Pierce Phosphatase Inhibitor Mini tablets; Thermo Scientific) on ice (10 min). Lysates were centrifuged at top speed in a microcentrifuge for 10 min. Before loading onto gels, samples were diluted with NuPAGE sample-reducing agent and NuPAGE lithium dodecyl sulfate (LDS) sample buffer (Life Technologies) and boiled (95°C, 5 min). Following protein separation on NuPAGE 4 to 12% polyacrylamide–bis-Tris gels (Life Technologies) and transfer to nitrocellulose, membranes were probed with primary antibodies. Primary antibodies used include monoclonal mouse anti-Mad2 (Santa Cruz Biotechnology; sc-374131), monoclonal mouse anti-actin (Sigma; A5441), monoclonal rabbit anti-BUB1 (Abcam; ab195268), monoclonal mouse anti-BUBR1 (Abcam; ab54894), monoclonal rabbit anti-BUB3 (Abcam; ab133699) and mouse anti-beta Tubulin (Invitrogen; A11126). Fluorescent dye-conjugated secondary antibodies (Li-Cor Biosciences) were used for infrared fluorescence-based detection (Odyssey CLX). Protein levels were quantified by measuring the relative fluorescence intensities of bands (normalized against actin) using Image Studio 2.1 software.

### Whole-Exome Sequencing and CNV Evaluation

The sequencing and bioinformatics analysis was performed at Indiana University Center for Computational Biology and Bioinformatics Core. The Agilent SureSelect XT2 Mouse All Exon kit was used to prepare the whole exome sequencing libraries for the paired normal and tumor samples. Four *Fancc-/-; Mad2+/-* mouse malignancies were sequenced (mouse IDs 501, 504, 520 and 576). The capture/target regions of the Agilent SureSelect design were downloaded from the Agilent website (https://earray.chem.agilent.com/suredesign/). These target regions were lift-overed (http://hgdownload.soe.ucsc.edu/admin/exe/linux.x86_64/liftOver) to mm10 coordinates since they were designed based on the mouse reference mm9. The resulting WES libraries were sequenced on Illumina HiSeq4000. ~100 million and ~200 million paired-end 75 bp reads were generated for the germline and tumor samples, respectively. The sequencing reads were first assessed for quality using FastQC (v.0.11.5, Babraham Bioinformatics, Cambridge, UK). Illumina adapter sequence and low-quality base calls were trimmed off the paired-end reads with Trim Galore (v0.4.3, http://www.bioinformatics.babraham.ac.uk/projects/trim_galore/). The remaining high-quality reads were next aligned to the mouse reference genome mm10 using BWA v7.15 (30). Duplicate reads were marked with Picard (https://broadinstitute.github.io/picard/) using the specific duplicate pixel distance parameter for HiSeq4000. Genome Analysis Toolkit (GATK v 3.7) (31) was subsequently applied for simultaneous indel realignment for each pair of the samples, to use MuTect 1 for somatic variant identification. GATK was used further to perform a base quality recalibration. MuTect 1, MuTect 2 (32) and Strelka (33) were used to identify somatic variants in the SureSelect capture regions. The variants identified from different tools were combined with GATK CombineVariants. To annotate the variants with ANNOVAR (34), we built a gene annotation database for mm10 according to the instructions from ANNOVAR. dbsnp142 was used for the annotation. The variants were also annotated with SnpEff (35). For detection of copy number variation (CNV), ExomeCNV (36) and CNVkit (37) were used. Depth coverage files were generated with GATK DepthOfCoverage for analysis with ExomeCNV. For the analysis with CNVkit, the blacklist regions for mm10 (downloaded from https://www.encodeproject.org/annotations/ENCSR636HFF/ by choosing mm10) were excluded in the access file.

### Evaluation of Predicted Variant Impact on Protein Function and Pathway Analysis

The Ensembl Variant Effect Predictor (VEP) and SIFT (Sorting Intolerant from Tolerant) bioinformatic algorithms (38, 39) were employed to evaluate genetic variants identified *via* WES. Variants (i) predicted by VEP to have a moderate or high impact, (ii) predicted by SIFT to be harmful to protein function and (iii) not present in reference mouse genomes as SNPs were selected for further analysis. The PANTHER bioinformatics strategy (40) was used for overrepresentation tests (GO biological process complete) using GO Ontology database (released 2018-08-09) with all *Mus musculus* genes serving as a reference. Fisher’s Exact with FDR (false discovery ratio) multiple test correction was used to determine the statistical significance of candidate pathway enrichment.

### Analysis of SAC Gene Mutation Frequency in FA-Wildtype and FA-Mutant Cancers

TCGA pan-cancer (PAN-CAN) dataset was accessed through UCSC Xena Browser (xenabrowser.net). Samples were categorized by either the presence (FA-mutant) or absence (FA-wildtype) of gene level non-silent mutations in the Fanconi Anemia pathway genes (BRCA1, BRCA2, BRIP1, ERCC4, FANCA, FANCB, FANCC, FANCD2, FANCE, FANCF, FANCG, FANCI, FANCL, FANCM, MAD2L2, PALB2, RAD51, RAD51C, RFWD3, UBE2T, SLX4, XRCC2). The frequency of gene level non-silent mutations in genes encoding the SAC proteins (MAD1L1, KNTC1, ZWILCH, ZW10, ZWINT, BUB1B, BUB3, BUB1, or MAD2L1) was evaluated in FA-mutant compared to FA-wildtype cancers.

### Statistical Analysis

All statistical analyses were performed using GraphPad Prism. The specific statistical test(s) performed for each experiment are detailed in the respective figure legends.

## Results

### *Fancc-/-; Mad2+/-* Mice Develop Early-Onset AML, Abnormal Hematopoiesis and Malignancies Throughout Life

**Figure 1:**
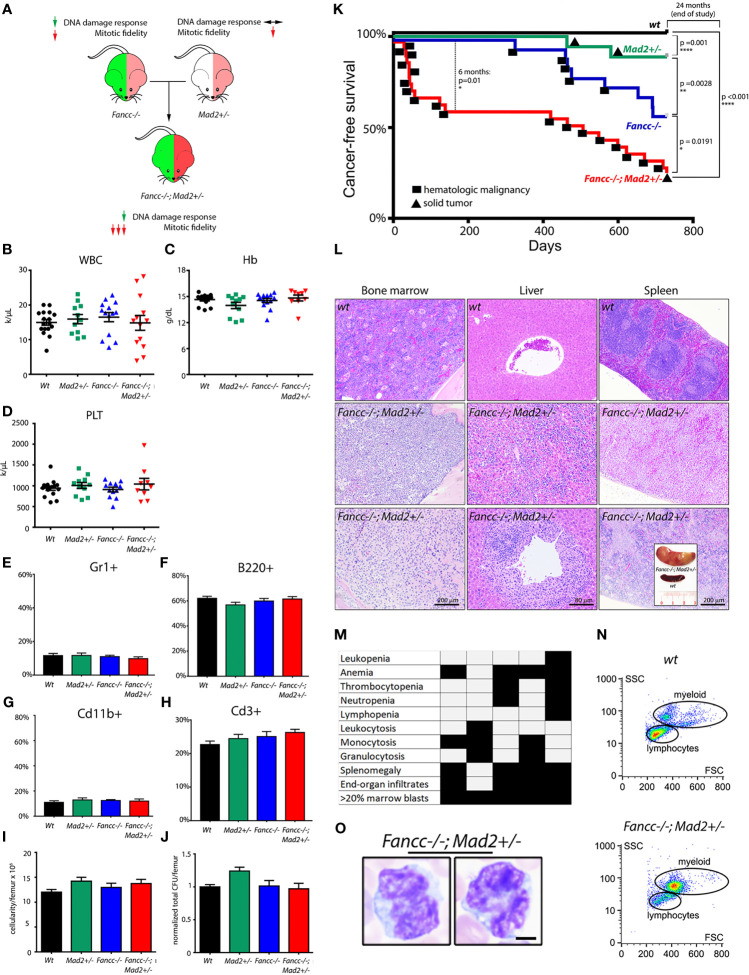
*Fancc-/-; Mad2+/-* mice suffer from premature death and abnormal hematopoiesis. **(A)** Experimental design. Mad2 heterozygosity in *Fancc-/-* background is hypothesized to exacerbate mitotic abnormalities but not DNA damage repair response. Normal total white blood count **(B)**, hemoglobin **(C)**, platelet count **(D)**, percentage of Cd11b+ and Gr1+ myeloid cells **(E, G)** and B220+ and Cd3+ lymphoid cells **(F, H)** in the peripheral blood of healthy-appearing *Fancc-/-; Mad2+/-* mice compared to age/sex-matched *wt*, *Mad2+/-* and *Fancc-/-* mice at 2-3 months of age. Well-appearing 16-week old *Fancc-/-; Mad2+/-* mice had the same bone marrow cellularity **(I)** and were able to form the same number of colonies in methylcellulose-based colony forming assays supplemented with progenitor growth factors (see *Methods*) **(J)** as age/sex-matched *wt*, *Fancc-/-* and *Mad2+/-* controls. Statistical analysis was performed by one-way ANOVA with Dunnett’s multiple comparison correction. No statistically significant differences were found. **(K)** Decreased cancer-free survival in *Fancc-/-; Mad2+/-* mice compared to *wt, Mad2+/-* and *Fancc-/-* age/sex-matched controls. Kaplan-Meier curves with p values determined by log-rank Mantel-Cox tests at 6-month and 24-month time points (n≥17 mice per genotype) are shown. Squares denote death due to hematopoietic malignancies; circles represent deaths from solid tumors. **(L)** Bone marrow, liver, and spleen infiltrates in representative *Fancc-/-; Mad2+/-* mice compared to *wt* controls. Insert (lower right) shows nodular splenomegaly in a representative leukemic *Fancc-/-; Mad2+/-* mouse. **(M)** Histopathological evidence of findings consistent with leukemia in five representative *Fancc-/-; Mad2+/-* mice. **(N)** flow cytometry demonstrating increased populations of large myeloid cells in a representative moribund *Fancc-/-; Mad2+/-* mouse. **(O)** Large myeloid blast cells, characterized by increased nucleus-to-cytoplasm ratio, irregular nuclei, and open chromatin from the peripheral blood. *p < 0.05, **p < 0.01, ****p < 0.0001.

*Fancc-/-;Mad2+/-* mice (**Figure 1A**) were born at a lower frequency than expected from Mendelian inheritance, consistent with previously reported *Fancc-/-* perinatal lethality (**Supplemental Table 1**). Similar to *Fancc-/-* mice*, Fancc-/-; Mad2+/-* mice surviving to term were smaller than *wt* littermates (36% and 22% average decrease in weight, respectively, relative to *wt*; **Supplemental Figure 1**) but free of gross deformities. Young *Fancc-/-; Mad2+/-* mice had normal hemoglobin and blood cell counts indistinguishable from *wt, Mad2+/-*, and *Fancc-/-* littermates (**Figures 1B–H**). Baseline bone marrow cellularity and hematopoietic colony-forming ability in *Fancc-/-;Mad2+-/-* mice were unremarkable (**Figures 1I, J** **)**. Consistent with our previous findings in *Fancc-/-* and work by others in *Mad2+/-* mice, neither *Mad2+/-* nor *Fancc-/-* mice developed early-onset cancer (6, 8, 26). We therefore reasoned that if error-prone chromosome segregation contributes to leukemogenesis in FA, then *Fancc-/-;Mad2+/-* mice should develop cancer at a greater frequency than age-matched *wt*, *Fancc*-/-, and *Mad2*+/- animals. Consistent with this hypothesis, *Fancc-/-; Mad2+/-* mice demonstrated decreased survival (p=0.01) at 6 months of age (**Figure 1K**). Moribund *Fancc-/-;Mad2+/-* mice exhibited extensive bone marrow infiltrates, peripheral cytopenia (10/10 examined mice), lymphadenopathy, splenomegaly with disrupted splenic architecture (8/10), and widespread hematopoietic infiltrates in the livers and other organs (8/10) (**Figures 1L, M** and **Supplemental Figures 2A–G**). This suggested that malignant hematopoiesis may be the cause of death in *Fancc-/-;Mad2+/-* mice. To further characterize these cancers, we followed the Bethesda criteria for hematopoietic malignancies and guidelines of AML/MDS diagnostics in mice (29, 41). The peripheral blood and bone marrow of moribund *Fancc-/-;Mad2+/-* mice contained large myeloid cells with increased nucleus-to-cytoplasm ratio, irregular nuclei, and open chromatin (**Figures 1N, O** **)**, similar to the AML/MDS seen in aging *Fancc-*/- mice (8). Immunophenotyping of blood and marrow consistently (6/6 moribund *Fancc-/-; Mad2+/-* mice) revealed increased population of immature Mac1+/Cd11b+ cells with elevated expression of c-kit but not B220 or Cd3 (**Supplemental Figures 2H–J**). These mice had normal blood counts four weeks before death. Together, these findings demonstrate that *Fancc*-/-;*Mad*2+/- mice rapidly develop lethal AML and are predisposed to develop cancer much earlier in life than either *Fancc*-/- or *Mad*2+/- mice.

**Figure 2:**
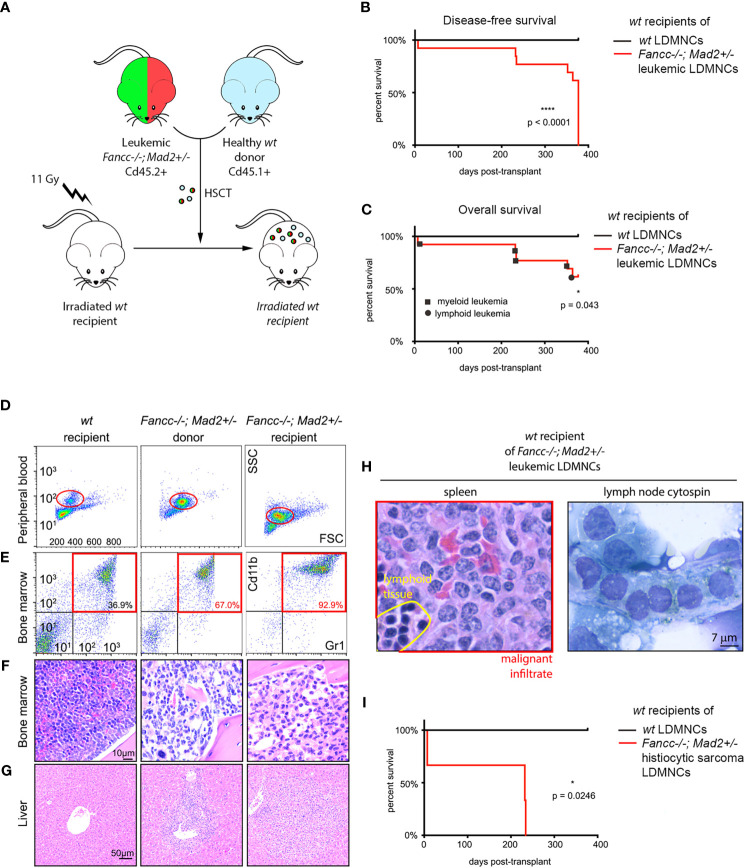
Lethal hematopoietic malignancy in *wt* mice transplanted with marrows of moribund *Fancc-/-; Mad2+/-* mice. **(A)** Experimental design of competitive hematopoietic cell transplantation. **(B)** Disease-free survival (p<0.0001) and **(C)** overall survival (p=0.043) of *wt* recipients transplanted with bone marrows of indicated genotypes, n =8 recipients of *wt* donor marrow and n=13 recipients of marrow from leukemic *Fancc-/-; Mad2+/-* donors. Statistical significance was calculated *via* log-rank Kaplan-Meier Mantel-Cox tests. **(D–G)** Comparison of peripheral blood and marrow flow cytometry, bone marrow and liver morphology in a *wt* mouse, *Fancc-/-; Mad2+/-* donor and *wt* recipient of *Fancc-/-; Mad2+/-* marrow. **(D, E)** Myeloid marker flow cytometry, **(F)** evaluation of bone marrow architecture and **(G)** liver infiltrates demonstrating similar immunophenotype and histopathologic findings in leukemic donor and recipients. **(H)** Malignant cell infiltration of lymph nodes and spleen in a representative moribund transplant recipient. **(I)** Survival of three recipient mice transplanted with bone marrow from a *Fancc-/-; Mad2+/-* donor mouse (p=0.0246). Statistical significance was determined with log-rank Kaplan-Meier Mantel-Cox tests. *p < 0.05, ****p < 0.0001.

We next evaluated the malignant potential of *Fancc-/-;Mad2+/-* hematopoietic cells through competitive hematopoietic stem cell transplant (HSCT) experiments, in which marrows from five moribund *Fancc-/-;Mad2+/-* mice were transplanted into lethally-irradiated *wt* recipients (**Figure 2A**). Engraftment was confirmed based on the presence of CD45.2+ LDMNCs in peripheral blood. Despite developing hematologic malignancies consistent with leukemia, cells from post-transplant peripheral blood samples obtained from the recipient mice of one moribund *Fancc-/-;Mad2+/-* donor mouse failed to demonstrate CD45.2 positivity. As such, data from these recipient mice were not included in our analyses. All mice transplanted with leukemic *Fancc-/-;Mad2+/-* marrows for which engraftment was confirmed by CD45.2+ LDMNCs in peripheral blood (recipients of 4/5 donor mice) had leukemia at necropsy one-year after transplant (**Figure 2B**), and 5/13 recipients of *Fancc-/-;Mad2+/-* leukemic marrows died within one-year post-HSCT (**Figure 2C**). Immunophenotype and histopathologic findings were the same in leukemic donor and recipients, as determined by myeloid marker flow cytometry (**Figures 2D, E** **)** and evaluation of bone marrow architecture **(** **Figure 2F** **)** as well as liver, spleen and lymph node infiltrates (**Figures 2G, H** **)**. Disease latency was similar in recipients of the same donor, but time to death in recipients differed depending on the donor (**Figure 2I**). Among those with confirmed engraftment, initial chimerism of *Fancc-/-; Mad2+/-* transplanted cells at 3 months post-transplant was varied in the peripheral blood of recipient mice (**Supplemental Figure 3**), suggesting that the transplanted cells arising from each donor have different engraftment potential. These results suggest that the leukemia arising in *Fancc-/-;Mad2+/-* mice are biologically heterogeneous but largely retain their malignant potential upon transfer into *wt* recipients.

To understand whether cancer predisposition in *Fancc-/-;Mad2+/-* mice differs from *Mad2+/-* and *Fancc-/-* mice (8, 26), we continued our phenotyping for two years. We found that an additional 7/20 (35%) of aging *Fancc-/-;Mad2+/-* mice died from lymphoid leukemias/lymphomas. These mice had chest and abdominal B220+ lymphadenopathy and hypocellular bone marrow infiltrated by blasts (**Supplemental Figure 4**). One *Fancc-/-* mouse developed sarcoma of the neck. At the end of the 2-year period, we examined all surviving mice through necropsy. We found malignancies in 4/7 (57%) of 2-year-old *Fancc-/-; Mad2+/-* survivors, including one myeloproliferative disorder, one large Ki67+, cytokeratin-negative retroperitoneal solid tumor with gland-like structures, and one lymphoma (**Supplemental Table 2**). We found one hepatocellular carcinoma and two solid tumors involving lungs in 3/14 aged *Mad2+/-* mice, as well as two lymphomas and two solid tumors in 4/12 aged *Fancc-/-* mice (**Supplemental Figure 5**). The incidence of malignancies was higher in surviving 2-year-old *Fancc-/-;Mad2+/-* mice compared to age-matched *wt* controls (1/17 mice) (p=0.0145). These findings expand the known patterns of malignancies for mice of these genotypes and, importantly, are consistent with the phenotype of human FA patients (8, 26).

### Non-Malignant Fancc-/-;Mad2+/- Cells Demonstrate Progressive Chromosomal Instability

**Figure 3:**
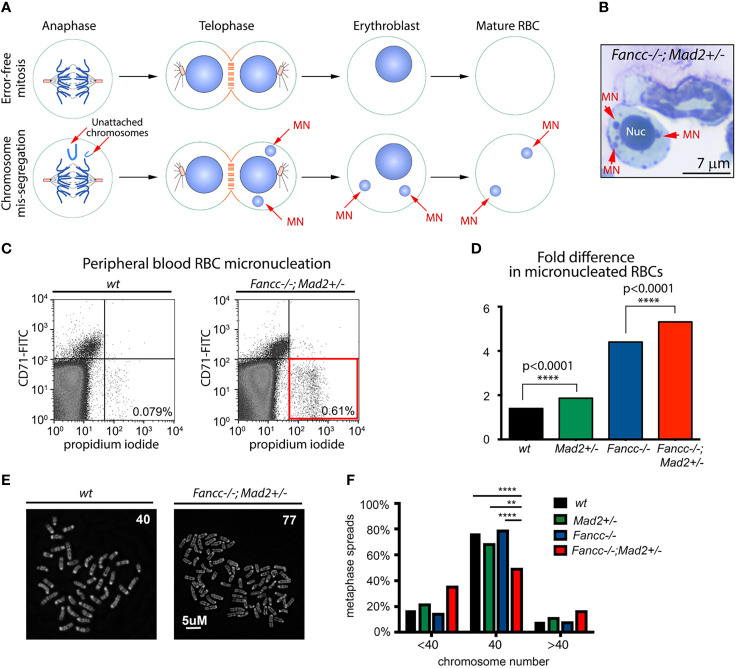
Spontaneous chromosomal instability in *Fancc-/-; Mad2+/-* mice. **(A)** Graphic illustrating the rationale of the quantitative red blood cell (RBC) micronucleation flow cytometry assay. Unlike normal nuclei, micronuclei (MN) arising from mitotic chromosome mis-segregation are not efficiently removed from maturing erythroblasts and therefore, DNA-positive mature RBCs signify abnormal chromosome sorting during erythropoiesis. **(B)** Example of a micronucleated cell in *Fancc-/-; Mad2+/-* bone marrow. Red arrows point to micronuclei (MNs). Nuc, nucleus. **(C, D)** Flow cytometry demonstrating an increased frequency of chromosome mis-segregation (p<0.0001) during *Fancc-/-; Mad2+/-* erythropoiesis compared to all other mouse genotypes. CD71 was used as a marker for immature peripheral blood red blood cells (RBCs). Frequencies of DNA-containing mature RBCs (CD71-, propidium iodide+) were statistically compared using Fisher’s exact test with n of at least 9 age-matched mice per genotype. **(E, F)** Karyotype demonstrating genomic instability in non-malignant *Fancc-/-;Mad2+/-* hematopoietic cells. Normal murine chromosome complement is 40, thus the presence of cells with <40 or >40 chromosomes is suggestive of aneuploidy. Statistical analysis was performed using Fisher’s exact test and Bonferroni *post-hoc* analysis (n=150 metaphase spreads per genotype, ****p < 0.0001, **p = 0.001).

While we demonstrate that *Fancc*-/-;*Mad*2+/- mice develop lethal AML and are predisposed to develop cancer earlier in life than both *Fancc*-/- and *Mad*2+/- mice, we also noted that all moribund *Fancc-/-;Mad2+/-* mice exhibited normal blood counts four weeks prior to death. We therefore asked whether *Fancc-/-;Mad2+/-* mice develop chromosomal instability (CIN) *in vivo* prior to the onset of cancer. We first used quantitative red blood cell (RBC) micronucleation flow cytometry assays (42) as previously described (8). Unlike normal nuclei, micronuclei arising from mitotic chromosome mis-segregation are not efficiently removed from maturing erythroblasts. Therefore, DNA-positive mature RBCs correspond to abnormal chromosome sorting during erythropoiesis (**Figures 3A, B** **)**. We observed a significant increase in micronucleated RBCs in peripheral blood of 12-month-old non-leukemic *Fancc-/-;Mad2+/-* mice compared to age-matched *Fancc-/-, wt* and *Mad2+/-* mice (**Figures 3C, D** **)**, further supporting our hypothesis that *Mad2*-haploinsufficiency and *Fancc* loss cooperate to potentiate chromosomal abnormalities. Karyotyping confirmed that non-malignant *Fancc-/-;Mad2+/-* hematopoietic cells and mouse embryonic fibroblasts (MEFs) are prone to aneuploidy (**Figures 3E, F** **)**, consistent with increased risk of genomic instability in *Fancc-/-;Mad2+/-* tissues.

### Error-Prone Mitosis Promotes Progressive Chromosomal Instability in Fancc-/-;Mad2+/- Mice

**Figure 4:**
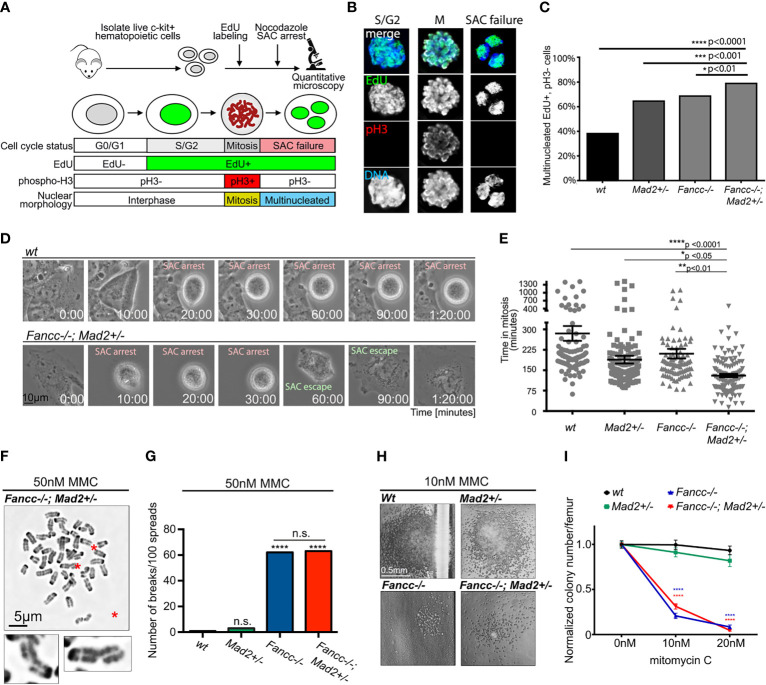
Exacerbation of SAC defect but not of DNA cross-linker hypersensitivity in *Fancc-/-; Mad2+/-* mice. **(A)** Graphic illustrating assay design for quantifying SAC impairment in primary low-density mononuclear hematopoietic cells (LDMNCs). Live c-kit+ cells were labeled with EdU and treated with 100 ng/mL nocodazole for 12 hours. Immunofluorescence microscopy quantifies cells that progressed through S-phase (EdU+) and escaped nocodazole-mediated SAC arrest to exit mitosis (pH3-) and undergo multi-nucleation (DNA). **(B)** Representative immunofluorescent images of c-kit+ cells in S/G2 phase (EdU+, pH3-, single nucleus), mitosis (M) (EdU+, pH3+, condensed chromosomes) and after SAC slippage (EdU+, pH3-, multi-nucleated). **(C)** Percentage of multinucleated Edu+, pH3- cells in *wt, Mad2+/-* and *Fancc-/-* and *Fancc-/-; Mad2+/-* c-kit+ cells, suggesting that SAC deficiency in *Fancc-/-; Mad2+/-* c-kit+ cells is more severe compared to *wt* (p<0.0001), *Mad2+/-* (p<0.001) and *Fancc-/-* (p<0.01) genotypes. *Fancc-/-* and *Mad2+/-* cells demonstrate an intermediate SAC defect. For each genotype, >200 cells were quantified, and results were compared by Fisher’s exact test. **(D)** Representative time-lapse video frames of *wt* and *Fancc-/-; Mad2+/*- BMDMs exposed to 2 μm taxol and imaged on a live imaging microscope at 2-minute intervals for 24 hours. SAC-arrested cells are round. SAC escape is followed by mitotic exit and flattening of multi-nucleated cells. **(E)** Evaluation of taxol-induced SAC arrest in *Fancc-/-; Mad2+/-* BMDMs. Time in mitosis (defined as the time from nuclear envelope breakdown to the time the nuclear envelope reforms) was recorded for at least 90 cells/genotype and analyzed by one-way ANOVA and Tukey’s *post-hoc* multiple comparisons test. The length of taxol-induced arrest in *Fancc-/-; Mad2+/*- BMDMs was significantly shorter compared to *wt* (p<0.0001), *Mad2+/-* (p<0.05) and *Fancc-/-* (p<0.01) genotypes. **(F)** Chromosome breaks (red asterisks) in *Fancc-/-; Mad2+/-* hematopoietic cells exposed to 50 nM MMC. Enlarged examples of fractured chromosomes are shown. **(G)** *Mad2* haploinsufficiency does not affect chromosome breakage in hematopoietic cells exposed to 50 nM MMC. Note that expected high frequency of MMC-induced chromosome breaks in *Fancc-/-* cells does not differ from *Fancc-/-; Mad2+/-* cells. Statistical significance was compared using Fisher’s exact test with at least 100 cells per genotype analyzed. ("ns" denotes "not significant"). **(H)** Images of representative hematopoietic colonies of indicated genotypes in MMC methylcellulose assays. **(I)** Evaluation of colony forming ability following MMC demonstrates that *Mad2* heterozygosity does not affect MMC hypersensitivity of *Fancc-/-* cells. *p < 0.05, **p < 0.01, ***p < 0.001, ****p < 0.0001.

Together, the above results suggest a role for error-prone mitoses as a driver of progressive chromosomal instability (CIN) in non-malignant *Fancc-/-;Mad2+/-* cells; however, an exacerbation of the underlying DDR defect could not be excluded. To better elucidate the mechanism driving progressive chromosomal instability in *Fancc-/-;Mad2+/-* mice, we characterized both mitotic and DDR defects and compared them to that of *wt, Mad2+/-*, and *Fancc-/-* mice. First, we quantified SAC impairment in primary low-density mononuclear hematopoietic cells (LDMNCs) of all genotypes as previously described (**Figures 4A–C**) **(** 4). The frequency of escape from nocodazole-induced SAC arrest was significantly greater in *Fancc-/-;Mad2+/-* cells compared to all other genotypes (*wt: p<0.0001, Mad2+/-: p<0.001)*, and *Fancc-/-: p <0.01)* (**Figure 4C**). As expected, *Mad2+/-* and *Fancc-/-* LDMNCs exhibited statistically significant increased incidences of escape from SAC arrest when compared to *wt*, but these were less severe than those seen in *Fancc-/-;Mad2+/-* LDMNCs. We then examined SAC integrity in primary bone marrow-derived macrophages (BMDMs) from all genotypes *via* time-lapse microscopy (**Figure 4D**), which revealed excessive escape from taxol-induced SAC arrest in *Fancc-/-;Mad2+/-* BMDMs (**Figure 4E**). We validated this finding through EdU/phospho-H3 fixed-imaging BMDM assays in which BMDMs of all genotypes (*wt*, *Fancc-/-*, *Mad2+/-* and *Fancc-/-; Mad2+/-*) were pulsed with EdU and treated with nocodazole for 12 hours to trigger the SAC (**Supplemental Figure 6**). To determine whether the *Fancc-/-;Mad2+/-* phenotype occurred secondary to exacerbation of the DNA repair defect characteristic of *Fancc-/-* cells, we compared the DNA damage repair defects among all genotypes through quantification of spontaneous and DNA cross-linker mitomycin C (MMC)-induced chromosome breakage as well as the colony-forming ability after MMC exposure (6). We observed a similar frequency of spontaneous chromosome breaks in *Fancc-/-;Mad2+/-* and *Fancc-/-* cells, and found that chromosome breakage in *Mad2+/-* cells was not increased compared to *wt* (**Supplemental Figure 7**). *Fancc-/-* and *Fancc-/-;Mad2+/-* hematopoietic cells accumulated similar numbers of MMC-induced chromosome breaks (**Figures 4F, G** **)** and demonstrated identical MMC hypersensitivity in methylcellulose progenitor assays (**Figures 4H, I** **)**. Since *Fancc* has been previously reported to prevent telomere attrition and increased sister-chromatid exchange (SCE), we evaluated telomeres and SCE in hematopoietic cells of all genotypes using telomere fluorescence *in-situ* hybridization (T-FISH) and SCE assays (43). We did not detect impaired telomere integrity or abnormal SCE in *Fancc-/-;Mad2+/-* cells (**Supplemental Figures 8A–C**). Together, these results indicate that increased chromosomal instability in *Fancc-/-;Mad2+/-* mice does not result from an exacerbation of the DDR defect nor due to failed telomere maintenance, but rather arises from SAC failure.

### Combined Loss of Fancc and Mad2 Compromises Mitotic Fidelity Through Exacerbation of Underlying SAC Defect

**Figure 5:**
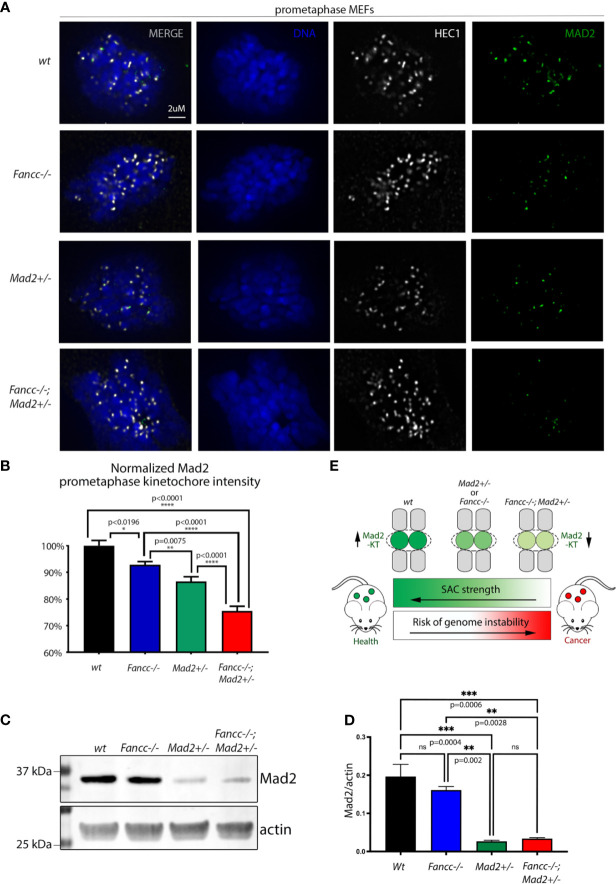
FANCC facilitates SAC function through recruitment of Mad2 to prometaphase KTs. **(A)** Representative immunofluorescent images and **(B)** quantification of endogenous MAD2 (green) signal intensity at MEF prometaphase KTs demonstrating decreased KT recruitment of MAD2 upon loss of *Fancc* (p<0.0196) compared to *wt*. Endogenous HEC1 (white) is used as a kinetochore marker. Endogenous MAD2 kinetochore immunofluorescence intensity was determined by deconvolution microscopy, quantified *via* Imaris and compared between genotypes using one-way ANOVA with Bonferroni *post-hoc* correction. Recruitment of MAD2 to prometaphase KTs is significantly decreased in *Fancc-/-; Mad2+/-* cells compared to *wt* (p<0.0001), *Mad2+/-* (p<0.0001) and *Fancc-/-* (p<0.0001) genotypes. **(C)** Western blotting of MEFs demonstrates that MAD2 expression is not altered upon loss of *Fancc [Fancc vs*. *wt:* (p=0.4789). and *Mad2+/- vs*. *Fancc-/-;Mad2+/-:* (p=0.9896)], with quantification of MAD2 signal normalized to actin shown in **(D)** ("ns" denotes "not significant"). **(E)** Summary schematic illustrating that *Fancc-/-; Mad2+/-* mice have decreased targeting of MAD2 to KTs, resulting in impaired SAC function and heightened genomic instability. *p < 0.05, **p < 0.01, ***p < 0.001, ****p < 0.0001.

FA-deficient cells consistently demonstrate features suggestive of abnormal cell division, and we and others have previously shown that the loss of FA-pathway proteins produces a phenotype consistent with SAC impairment (4, 44–49). Above, we have confirmed the presence of an underlying SAC defect in *Fancc-/-* cells and demonstrated that this defect is exacerbated in *Fancc-/-; Mad2+/-* cells. Previous data suggests that the SAC does not function as an “all or nothing” response, but rather as a rheostat that can be engaged to various degrees (9). SAC strength is known to be dependent on the amount of MAD2 recruited to the kinetochore such that depletion of kinetochore-bound MAD2 below a certain threshold causes functional compromise of the SAC, allowing cells to exit mitosis prematurely (9). To assess the role of FANCC in MAD2 localization, we employed HEC1 as a kinetochore marker and quantified the fluorescence signal intensity of MAD2 at prometaphase kinetochores *via* immunofluorescence microscopy in *wt*, *Fancc-/-, Mad2+/-*, *and Fancc-/-;Mad2+/-* mouse embryonic fibroblasts (MEFs). We validated our approach by demonstrating the expected decrease in MAD2 at prometaphase kinetochores in *Mad2+/-* cells. We found that MAD2 intensity was significantly decreased at *Fancc*-/- kinetochores relative to *wt* kinetochores, and the amount of MAD2 on *Fancc-/-;Mad2+/-* prometaphase kinetochores was more significantly decreased than both *Mad2+/-* and *Fancc-/-* MEFs (**Figures 5A, B** **)**. Western blot analysis revealed comparable levels of total MAD2 protein in *Fancc-/-* and *wt* cells (p=0.4789), indicating that reduced accumulation of MAD2 at *Fancc-/-* kinetochores was not due to reduced protein expression (**Figures 5C, D** **)**. Similarly, MAD2 protein expression was not significantly different between *Mad2+/- and Fancc-/-;Mad2+/-* (p=0.9896). Western blot analysis of SAC proteins BUB1, BUB1B and BUB3 also demonstrated comparable expression across all four genotypes (**Supplemental Figure 9**). These results support a model in which loss of *Fancc* impairs kinetochore recruitment of MAD2, such that further depletion of the kinetochore-bound pool of MAD2 through heterozygous deletion of *Mad2* results in a compromised SAC that is insufficient to ensure mitotic fidelity. (**Figure 5E**).

### Fancc-/-; Mad2+/- Malignancies Are Characterized by Mitotic Infidelity and Chromosomal Abnormalities Implicated in Human FA-Associated Malignancies

**Figure 6:**
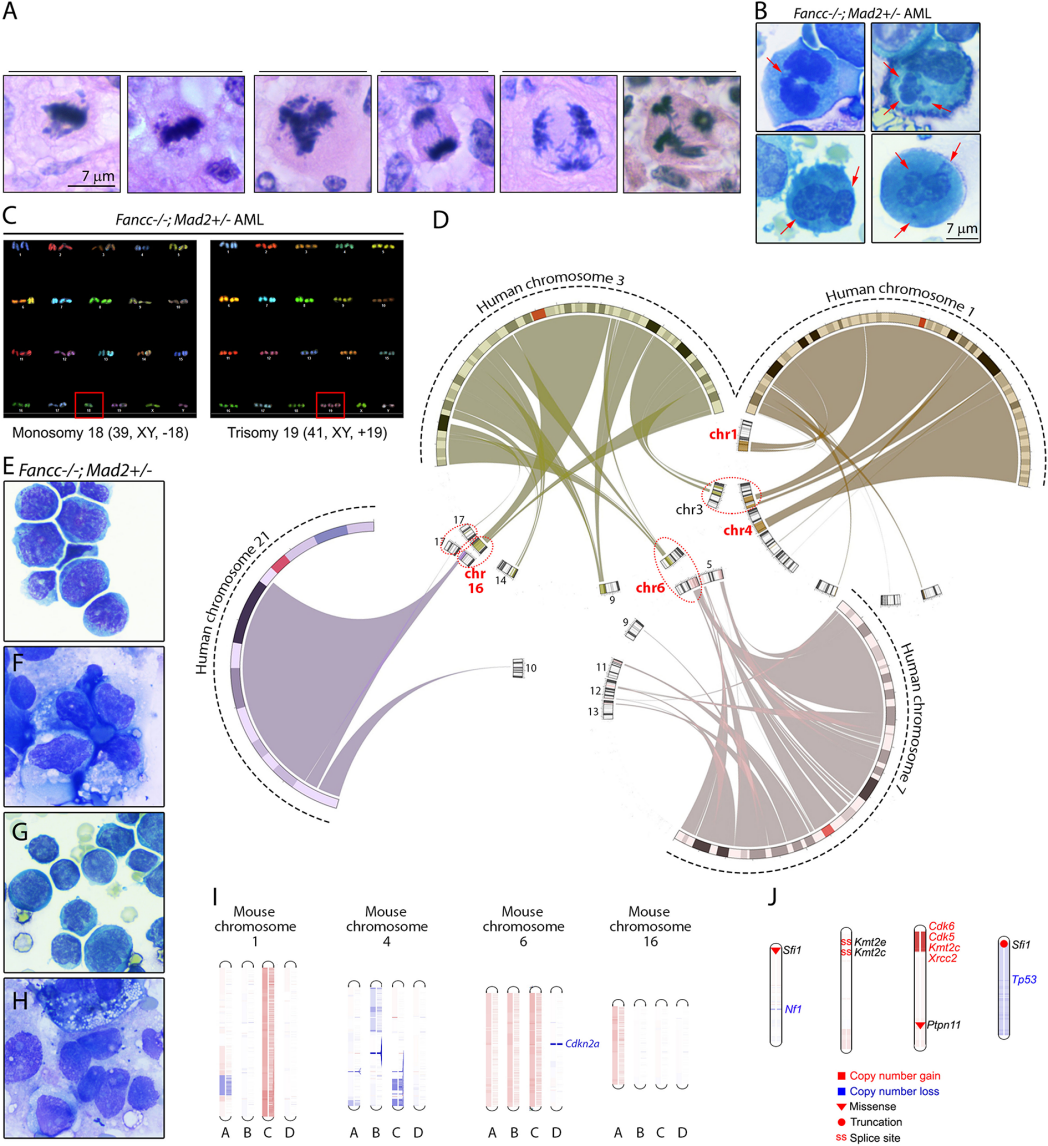
Mitotic infidelity and genomic lesions in Fancc-/-; Mad2+/- malignancies. **(A)** Representative images of abnormal mitotic figures in Fancc-/-; Mad2+/- hematopoietic malignancies *in situ* and **(B)** in bone marrow cytospins. Red arrows indicate lagging anaphase chromosomes, multi-nucleation and bi-nucleation in bone marrow cells isolated from Fancc-/-; Mad2+/- AML mice. **(C)** SKY analysis of Fancc-/-; Mad2+/- leukemia shows clonal hematopoiesis with whole-chromosome gains and losses. **(D)** Circular synteny map shows mapping of human chromosomes 1, 3, 7 and 21, whose changes had been implicated in FA MDS/AML in previous studies, to mouse chromosomes. Mouse chromosomes containing regions of more than one of the four human chromosomes are highlighted in circles. **(E–H)** Representative images of Fancc-/-; Mad2+/- malignancies. **(I)** Whole-exome sequencing of four Fancc-/-; Mad2+/- malignancies revealed copy-number variations of mouse chromosomes 1, 4, 6 and 16. Unique Fancc-/-; Mad2+/- malignancies are represented by letters A-D. Red denotes amplifications and blue represents copy number losses. Additional somatic variants of genes implicated in human cancer and genome stability identified by WES in Fancc-/-; Mad2+/- malignancies are shown in **(J)**. Types of mutations are shown in the visual legend.

Given the above results, we suspected that the progressive chromosome instability resulting from increased error-prone mitoses played a critical role in the malignant transformation of *Fancc-/-;Mad2+/-* cells. Indeed, we found abundant abnormal mitoses in all *Fancc-/-;Mad2+/-* AMLs *in situ* and marrow cytospins (**Figures 6A, B** **)**. Further, SKY chromosome-painting confirmed karyotype instability in a moribund *Fancc-/-;Mad2+/-* AML mouse (**Figure 6C**). To explore the similarities between FA MDS/AML in humans and mice, we first mapped the chromosomes frequently mutated in human FA MDS/AML (chromosomes 1, 3, 7 and 21) (50) to the mouse genome. Our comparative genomics analysis (51, 52) mapped the relevant human chromosome regions to mouse chromosomes 3, 6 and 16; furthermore, portions of human chromosome 1 map to mouse chromosomes 1 and 4, and portions of human chromosome 3 are represented by mouse chromosome 9 (**Figure 6D**). We next examined genomic instability in four *Fancc-/-;Mad2+/-* malignancies (**Figures 6E–H**) through whole-exome sequencing (WES) coupled with copy number variation (CNV) analysis. We noted copy number abnormalities of chromosome 16 in 4/4 analyzed malignancies; chromosome 1 was affected in 2/4 and chromosome 6 in 1/4 malignancies (**Figure 6I**). PANTHER pathway analysis of WES results in four *Fancc-/-;Mad2+/-* malignancies detected enrichment of variants within genes involved in hematopoiesis, mitosis, apoptosis, growth factor signaling, and stress response (**Supplemental Table 3**) (40). We also noted acquired splicing variants within *Kmt2e* and *Kmt2c* MLL (mixed lineage leukemia) lysine methyltransferase genes, and loss of *Tp53* locus (**Figure 6J** and data not shown). These studies provide initial insights into the somatic evolution of hematopoietic genome instability in *Fancc-/-; Mad2+/-* mice as they progress towards MDS/AML and suggest features that are similar to human disease.

## Discussion

**Figure 7:**
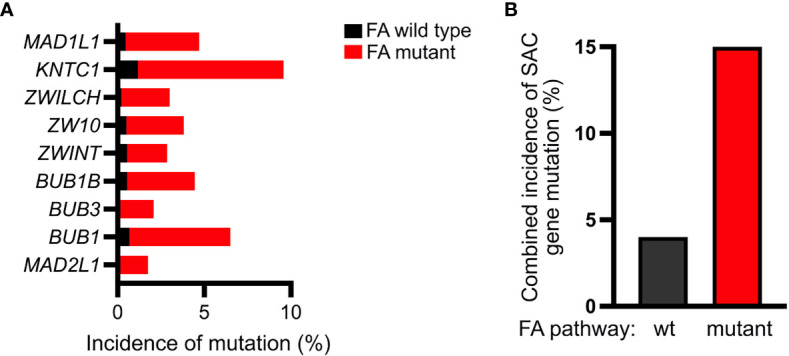
FA-deficient cancers exhibit a higher incidence of mutations within genes encoding SAC proteins. **(A)** Analysis of the TCGA pan-cancer dataset acquired through UCSC Xena browser (xenabrowser.net) demonstrating increased frequency of mutations within genes encoding the indicated SAC proteins in FA-mutant (red) compared to FA-wildtype (black) cancers **(B)** Accordingly, relative to FA-wildtype cancers (black), FA-mutant (red) cancers exhibit a nearly 4-fold increase in the mutation frequency of one or more of the following SAC genes: MAD1L1, KNTC1, ZWILCH, ZW10, ZWINT, BUB1B, BUB3, BUB1, or MAD2L1.

Error-prone mitosis occurring secondary to disruption of the SAC can facilitate the acquisition of genetic alterations necessary to promote tumorigenesis (49, 53). Under physiologic conditions, the SAC protects cells from chromosomal instability and aneuploidy by ensuring accurate chromosome segregation (54, 55). While somatic mutations of genes encoding SAC proteins are reported infrequently, they occur at a nearly 4-fold increased frequency in FA pathway deficient cancers compared to FA pathway proficient cancers (**Figure 7**). Additionally, disruption of SAC protein function is widely reported in a variety of cancers and non-mutational alterations in SAC proteins, including MAD2, have specifically been reported in AML/MDS (11, 16, 17, 56–59).

The role of SAC dysfunction in driving tumorigenesis has previously been investigated utilizing mouse strains deficient in BUB1B, BUB3, and MAD2. These studies demonstrated that reduced expression of *Bub3 (* 60, 61) and *Bub1b (* 62) did not significantly increase the tumor incidence, and haploinsufficiency of *Mad2 (* 26) caused only a mild increase in spontaneous tumors. Notably, a shared feature of the of these models is that the tumors developed only in old age, suggesting that loss of SAC protein function alone is not sufficient to drive tumorigenesis and that cells must acquire additional “hits” to undergo transformation.

Likewise, mice deficient in key FA pathway proteins, including *Fancc*, *Fancg*, *Fanca*, and *Fancd2*, also do not develop cancers until later in life (14 months of age), and only *Fancc*-/- mice are known to develop AML/MDS in old age (5, 8). Fanconi anemia confers a several hundred-fold increased risk of developing AML/MDS compared to the general population, and treatment of these patients is challenging due to cytotoxic chemotherapeutic hypersensitivity (1). As such, the development of mouse models of spontaneous AML associated with loss of core FA genes is crucial for preclinical studies and therapeutics development (63). Several genetic strategies to accelerate the development of AML in FA core complex-deficient mice have previously been explored; however, while these mice are known to develop solid tumors and lymphoid malignancies, they do not develop early-onset AML/MDS (64–70).

Previous studies suggest that FA protein loss and SAC inactivation cooperate to promote malignant transformation (1, 10–15). Thus, we hypothesized that SAC compromise would be sufficient to drive leukemogenesis in the setting of FA pathway dysfunction. The strength of the SAC is directly correlated with the amount of MAD2 associated with prometaphase kinetochores, which allowed us to fine-tune the frequency of mitotic errors in *Fancc-/-* mice by introducing *Mad2* heterozygosity (9). The resultant *Fancc-/-; Mad2+/-* mice developed early-onset AML and exhibited abnormal hematopoiesis characterized by error-prone mitoses and progressive chromosomal instability preceding malignant transformation. We found that overt leukemogenesis in *Fancc-/-; Mad2+/-* mice is associated with increased mitotic infidelity secondary to compromise of the spindle assembly checkpoint (SAC) and that loss of *Fancc* impairs recruitment of MAD2 to prometaphase kinetochores. Accordingly, we show that leukemogenesis in *Fancc-/-; Mad2+/-* mice does not arise from exacerbation of the underlying DNA damage repair defect, but rather as a result of SAC insufficiency due to decreased MAD2 localization to prometaphase kinetochores. Furthermore, analysis of *Fancc-/-; Mad2+/-* murine leukemias through WES, CNV and SKY suggests the presence of genetic lesions that overlap with acquired chromosomal changes reported in human FA AML/MDS (50, 71, 72). Importantly, we noted copy number alterations in chromosomes that map to human chromosomes 1, 3, and 7, which are frequently affected in FA patients with MDS and AML (73). These findings suggest that leukemogenesis in this murine model reflects the molecular pathogenesis that underlies malignant transformation in FA patients.

Here, we have presented a novel murine model that reflects the AML-prone phenotype of human FA patients and provides insight into the role of mitotic abnormalities as drivers of leukemogenesis in the setting of FA pathway deficiency. The propensity of our *Fancc-/-; Mad2+/-* mice to develop early-onset AML provides a valuable tool for the development of evidence-driven therapies to optimize outcomes and decrease therapy-related toxicities in patients with FA who suffer from AML/MDS.

## Data Availability Statement

The datasets presented in this study can be found in online repositories. The names of the repository/repositories and accession number(s) can be found below: NCBI Sequence Read Archive (SRA) under accessions SAMN21889475, SAMN21889476, SAMN21889477, SAMN21889478, SAMN21889479, SAMN21889480, SAMN21889481, SAMN21889482.

## Ethics Statement

The animal study was reviewed and approved by Institutional Animal Care and Use Committee at Indiana University.

## Author Contributions

DE and ES performed experiments, analyzed data, and prepared the manuscript. DM analyzed data and prepared manuscript. ZA, AS, and YH performed experiments and contributed to data analysis. K-KC performed experiments and analyzed mouse CNV/WES data. P-JC and YL performed and analyzed telomere quantification and SCE experiments. DWC and HB provided intellectual input and assisted in preparation and editing of the manuscript. RS and GC provided intellectual input. LJ and JY helped with mouse colony maintenance. GN conceived the study and assisted in manuscript preparation. All authors contributed to the article and approved the submitted version.

## Funding

This work was supported by the NIH R01-HL132921-01A1 award (DWC), St. Baldrick’s Foundation Scholar award (GN), Heroes Foundation (GN), the Bone Marrow Failure Research Fund at Riley Children’s Foundation (GN), NIH T32 HL007910 “Basic Science Studies on Gene Therapy of Blood Diseases” grant (ES), NIH Diversity Supplement 3R01HL132921-03S1 (ES), and NCI 1F30CA200227-01A1 fellowship (DE).

## Conflict of Interest

The authors declare that the research was conducted in the absence of any commercial or financial relationships that could be construed as a potential conflict of interest.

## Publisher’s Note

All claims expressed in this article are solely those of the authors and do not necessarily represent those of their affiliated organizations, or those of the publisher, the editors and the reviewers. Any product that may be evaluated in this article, or claim that may be made by its manufacturer, is not guaranteed or endorsed by the publisher.
