# Supplementary material for: Mitotic Errors Promote Genomic Instability and Leukemia in a Novel Mouse Model of Fanconi Anemia
Source: Front Oncol. 2021 Nov 5;11:752933. doi: 10.3389/fonc.2021.752933 (PMC8602820; doi:10.3389/fonc.2021.752933)
Supplement: Supplementary file 1 [file DataSheet_1.pdf]

Supplementary Material

Supplementary Data

Parental genotypes: *Fancc+/-* x *Fancc+/-; Mad2+/-*			
**Genotype**	**Expected number of mice (%)**	**Observed number of mice (%)**	**Chi-square p-value**
Wt	73 (12.5%)	90 (15.43%)	0.1510
Mad2+/-	73 (12.5%)	70 (12.01%)	0.7888
Fancc-/-	73 (12.5%)	44 (7.55%)	**0.0047**
Fancc-/-;Mad2+/-	73 (12.5%)	37 (6.35%)	**0.0003**
Fancc+/-	145 (25%)	174 (29.85%)	0.0567
Fancc+/-;Mad2+/-	145 (25%)	167 (28.64%)	0.2013
**Total offspring**	**582**	**582**	

Supplementary Table 1. *Fancc-/-; Mad2+/-* mice are born at frequencies comparable to *Fancc-/-* mice. *Fancc-+/-* were bred to *Fancc+/-; Mad2+/-* mice and we genotyped the resulting 582 pups for analysis. We show expected and observed numbers and percentage of animals for each genotype. We analyzed results with chi-square test followed by Bonferroni post-hoc correction. Statistically significant *p* values are shown in **red**.

Mouse ID	Genotype	Days lived	Malignancy
147	*Fancc-/-; Mad2+/-*	EOS	None
383	*Fancc-/-; Mad2+/-*	EOS	solid tumor
588	*Fancc-/-; Mad2+/-*	EOS	None
576	*Fancc-/-; Mad2+/-*	EOS	acute myeloid leukemia
440	*Fancc-/-; Mad2+/-*	EOS	None
501	*Fancc-/-; Mad2+/-*	EOS	lymphoid leukemia/lymphoma
520	*Fancc-/-; Mad2+/-*	EOS	solid tumor
887	*Fancc-/-; Mad2+/-*	33	acute myeloid leukemia
846	*Fancc-/-; Mad2+/-*	36	acute myeloid leukemia
204	*Fancc-/-; Mad2+/-*	139	acute myeloid leukemia
233	*Fancc-/-; Mad2+/-*	420	acute myeloid leukemia
99	*Fancc-/-; Mad2+/-*	670	acute myeloid leukemia
504	*Fancc-/-; Mad2+/-*	465	acute myeloid leukemia/histiocytic sarcoma
251	*Fancc-/-; Mad2+/-*	44	unknown
637	*Fancc-/-; Mad2+/-*	42	unknown
704	*Fancc-/-; Mad2+/-*	42	lymphoid leukemia
415	*Fancc-/-; Mad2+/-*	506	lymphoid leukemia
430	*Fancc-/-; Mad2+/-*	548	lymphoid leukemia
145	*Fancc-/-; Mad2+/-*	622	lymphoid leukemia
420	*Fancc-/-; Mad2+/-*	599	lymphoid leukemia/lymphoma
164	*Fancc-/-; Mad2+/-*	730	lymphoid leukemia/lymphoma
192	*Fancc-/-; Mad2+/-*	48	unknown
711	*Fancc-/-; Mad2+/-*	124	acute myeloid leukemia
141	*Fancc-/-; Mad2+/-*	720	solid tumor
223	*Fancc-/-; Mad2+/-*	57	unknown
1037	*Fancc-/-; Mad2+/-*	28	leukemia

Supplementary Table 2. Cancer predisposition in *Fancc-/-; Mad2+/-* mice. All mice were sacrificed with spleen, liver, bone marrow (femur cross-sections in addition to cytospin), and any grossly abnormal tissues were evaluated in a blinded fashion by at least two investigators. Myeloid and lymphoid malignancies were distinguished using flow cytometry and/or immunohistochemistry with myeloid (Gr-1; Mac-1; Mpo) and lymphoid (Cd3; Cd4; B220) markers in addition to a blinded review of histology slides, bone marrow cytospins, and peripheral blood smears. In mice labeled as "unknown", the final diagnosis was not established due to sample quality. EOS: end of study.

GO biological process complete	Observed variants	Expected variants	+/-	Fold enrichment	p value	FDR
**Apoptosis**						
hepatocyte apoptotic process (GO:0097284)	6	0.71	+	8.44	2.90E-04	3.22E-02
**Hematopoietic cell differentiation and function**						
megakaryocyte development (GO:0035855)	8	0.98	+	8.13	3.44E-05	6.86E-03
natural killer cell activation involved in immune response (GO:0002323)	11	1.69	+	6.49	6.72E-06	1.83E-03
B cell proliferation (GO:0042100)	14	2.57	+	5.45	2.14E-06	7.39E-04
T cell activation involved in immune response (GO:0002286)	14	3.94	+	3.56	1.29E-04	1.77E-02
**Mitosis**						
mitotic DNA integrity checkpoint (GO:0044774)	12	3.5	+	3.43	5.10E-04	4.99E-02
microtubule organizing center organization (GO:0031023)	16	4.97	+	3.22	1.24E-04	1.74E-02
**Growth factor signaling**						
positive regulation of peptidyl-serine phosphorylation of STAT protein (GO:0033141)	11	1.53	+	7.19	3.04E-06	1.03E-03
cellular response to epidermal growth factor stimulus (GO:0071364)	9	2.02	+	4.45	5.08E-04	5.00E-02
**Stress response**						
response to exogenous dsRNA (GO:0043330)	13	3.12	+	4.17	5.50E-05	9.49E-03
DNA conformation change (GO:0071103)	24	9.57	+	2.51	1.30E-04	1.78E-02

Supplementary Table 3. Whole-exome sequencing reveals enrichment of somatic variants within genes regulating apoptosis, hematopoiesis, mitosis, growth factor signaling and stress response in *Fancc-/-; Mad2+/-* malignancies. Genetic variants were analyzed via PANTHER over-representation test and organized into master signaling pathways. Top five signaling networks are shown. Expected variants and fold enrichment were calculated by PANTHER based on all mouse genes in the database and annotated against the GO ontology database. FDR: false discovery ratio.

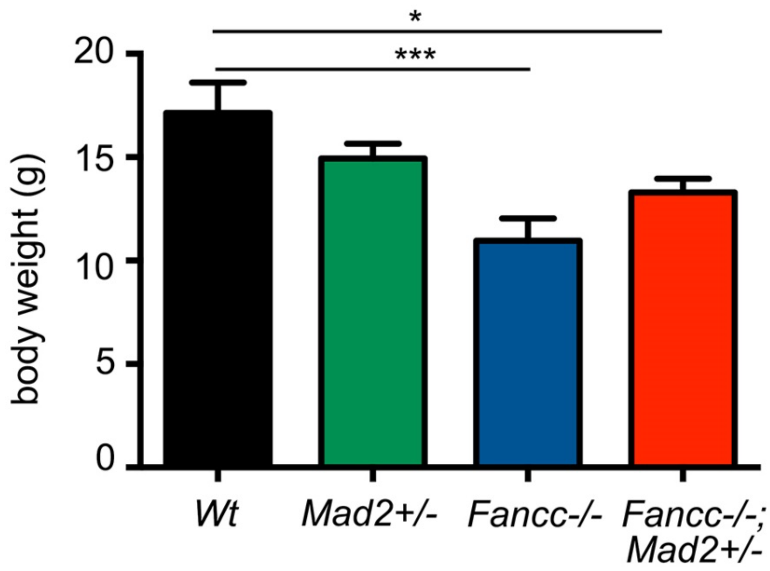

Supplementary Figure 1. *Fancc-/-; Mad2+/-* mice and *Fancc-/-* mice are smaller than age/sex-matched *wt* littermates. The weight of 4-week old male mice (n>=9 mice per genotype) was compared by one-way ANOVA with Tukey's multiple comparisons test. *** p<=0.001; * p<=0.05.

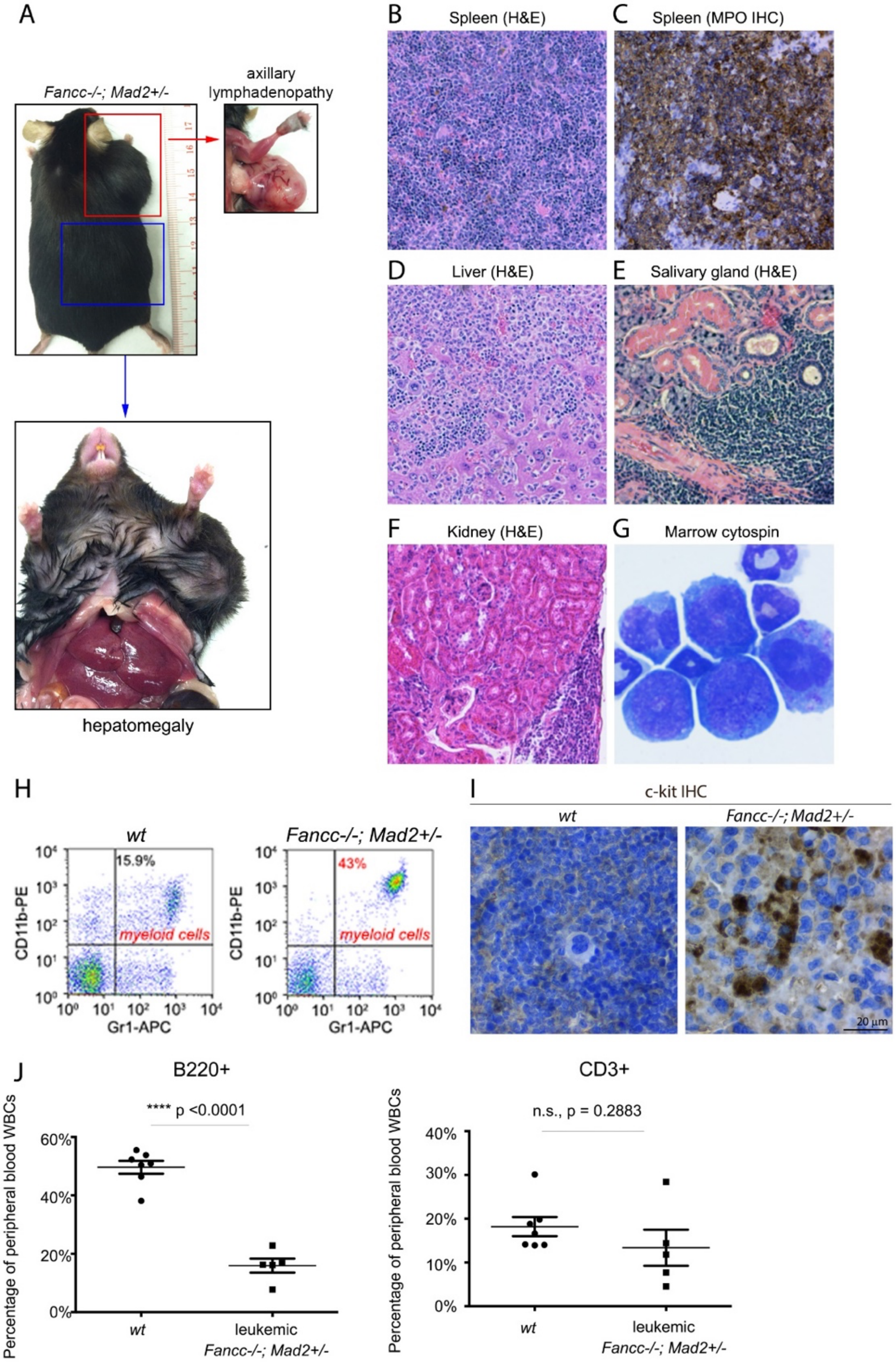

Supplementary Figure 2. Myeloproliferative disorders in *Fancc-/-; Mad2+/-* mice. Representative *Fancc-/-; Mad2+/-* mouse with gross axillary lymphadenopathy and hepatosplenomegaly (**A**). Disrupted spleen architecture (**B**) with Mpo+ infiltrates (**C**); liver (**D**) and salivary gland (**E**) infiltrates; sub-capsular kidney infiltrate (**F**) and a cluster of bone marrow granular myeloid cells next to a prophase cell (**G**) in *Fancc-/-; Mad2+/-* mice. (**H**) Flow cytometry depicting increased Gr1+ Cd11+ myeloid cells in LDMNCs isolated from peripheral blood of a moribund *Fancc-/-; Mad2+/-* mouse compared to an age-matched *wt* control. (**I**) Increased c-kit positivity by immunohistochemistry in *Fancc-/-; Mad2+/-* bone marrow infiltrated with large myeloid blasts. (**J**) Flow cytometry of the percentage of LDMNCs from peripheral blood of moribund *Fancc-/-; Mad2+/-* mice expressing

lymphoid makers B220 (left) and CD3 (right) compared to age-matched controls. Statistics obtained via student's t-test, n= 5-7 mice per genotype.

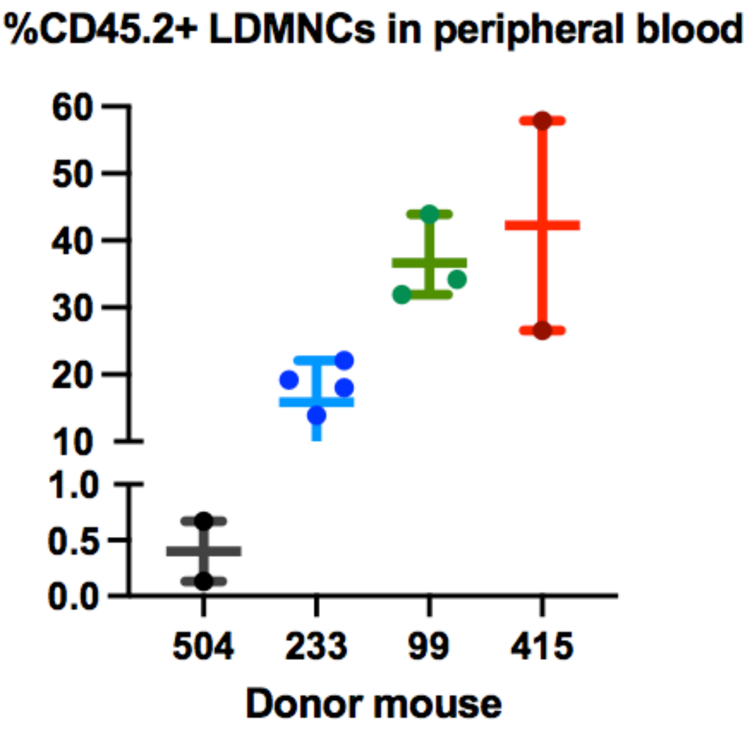

Supplementary Figure 3. **Percentage of CD45.2+ LDMNC engraftment as measured in peripheral blood of recipient mice transplanted with CD45.2+ LDMNCs from *Fancc-/-; Mad2+/-* leukemic donors.** Donor LDMNCs from *wt* or *Fancc-/-;Mad2+/-* (C57Bl/6J, CD45.2+) mice were transplanted with competitor cells from *wt* donor mice (BoyJ, CD45.1+) into healthy irradiated recipients as described (see methods). Percentage of CD45.2 positivity in LDMNCs from peripheral blood of *wt* recipients of each *Fancc-/-; Mad2+/-* donor mouse (donor identities of 4 *Fancc-/-; Mad2+/-* leukemic mice shown along x axis) was assessed at about 3 months post-transplant. Each data point on the graph is representative of one recipient mouse post-transplant, bars depict **the range**.

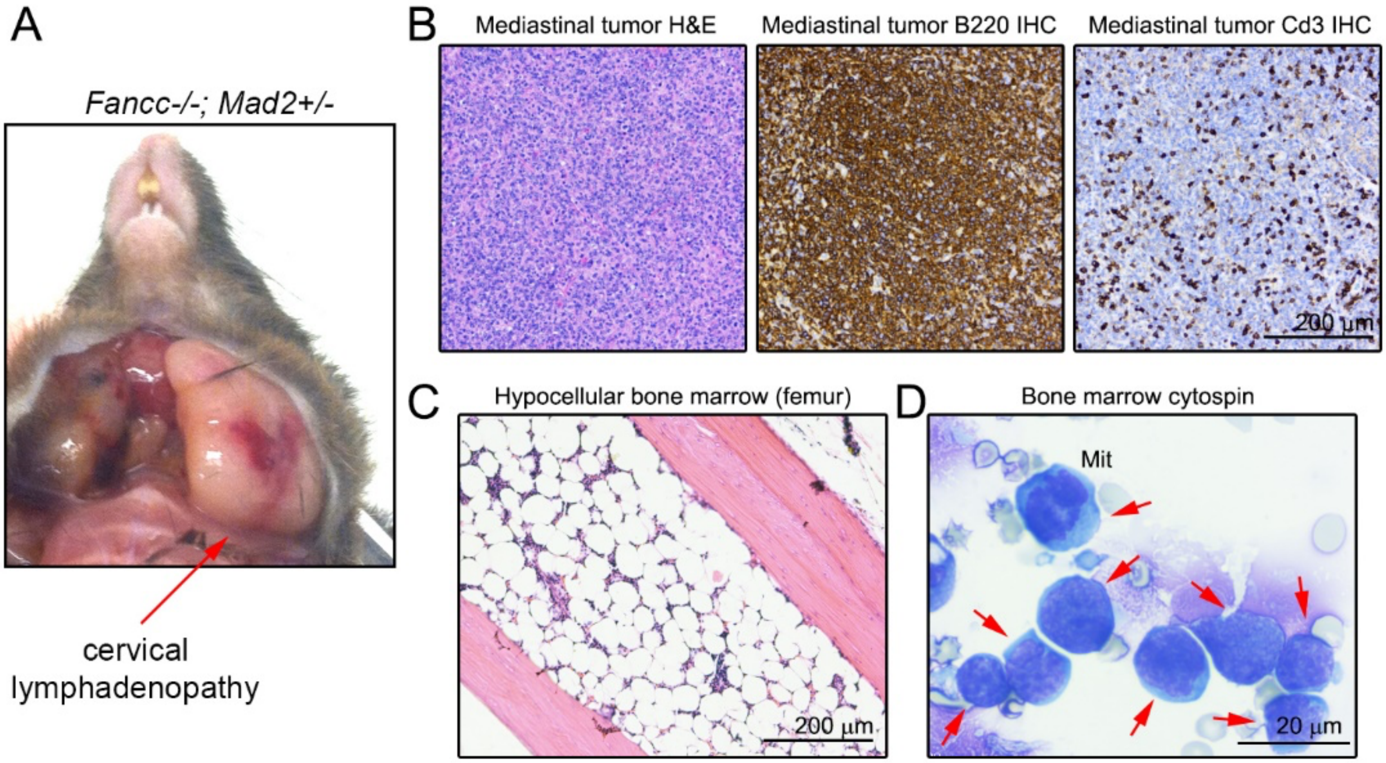

Supplementary Figure 4. Lymphoproliferative disorders in *Fancc-/-; Mad2+/-* mice. Representative *Fancc-/-; Mad2+/-* mouse with a large cervical conglomerate of lymph nodes (**A**). Mediastinal tumor of large cells strongly expressing B220 with scattered Cd3+ cells (**B**). Hypocellular bone marrow (**C**) with hematopoiesis replaced by blasts with the high nucleus-to-cytoplasm ratio (red arrows); note a mitotic cell with scattered chromosomes (**D**).

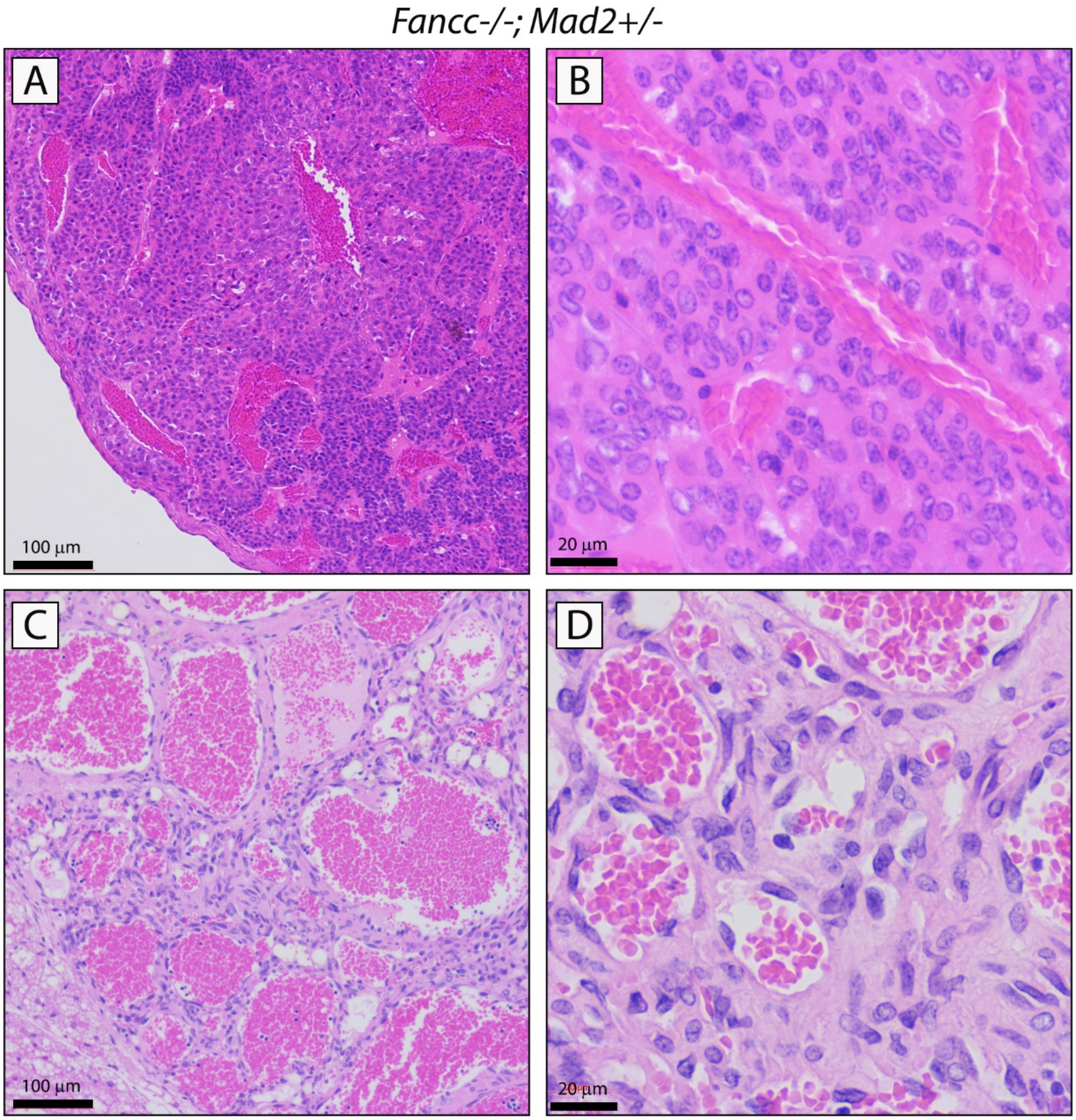

Supplementary Figure 5. Solid tumors in *Fancc-/-; Mad2+/-* mice. Colon carcinoma (**A, B**) and highly vascularized sarcoma of the neck (**C, D**) are shown.

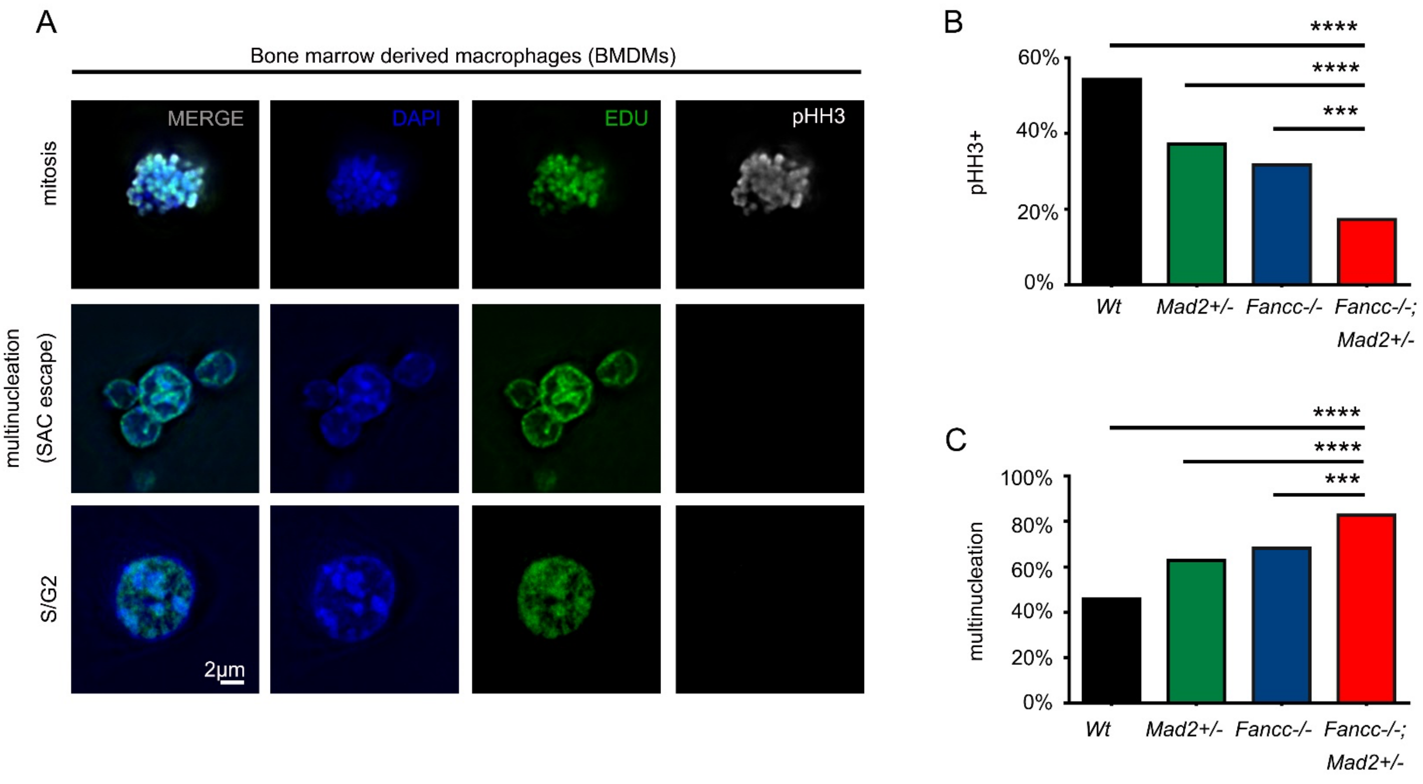

Supplementary Figure 6. Impaired SAC arrest in proliferating *Fancc-/-; Mad2+/-* BMDMs. BMDMs of all genotypes (*wt*, *Fancc-/-*, *Mad2+/-* and *Fancc-/-; Mad2+/-*) were pulsed with EdU, treated with nocodazole for 12 hours to trigger the SAC, fixed and stained with phospho-H3 antibody and Hoechst 33342 to highlight mitotic chromosomes and DNA, respectively. Representative images of mitotic (EdU+, pH3+, visible chromosomes) BMDMs compared to BMDMs that escaped SAC arrest (EdU+, pH3-, multinucleation) and BMDMs fixed in S/G2 before mitotic entry (EdU+, pH3-, single nucleus) are shown in **A**. Quantification of pH3-positivity and multinucleation are shown in **B** and **C**. Note decreased pHH3+ cells and increased multinucleation in *Fancc-/-; Mad2+/-* BMDMs compared to all other genotypes, consistent with decreased SAC fidelity. At least 200 cells per genotype were analyzed with results compared via Fisher's exact test (*** = $p<0.001$; **** = $p<0.0001$).

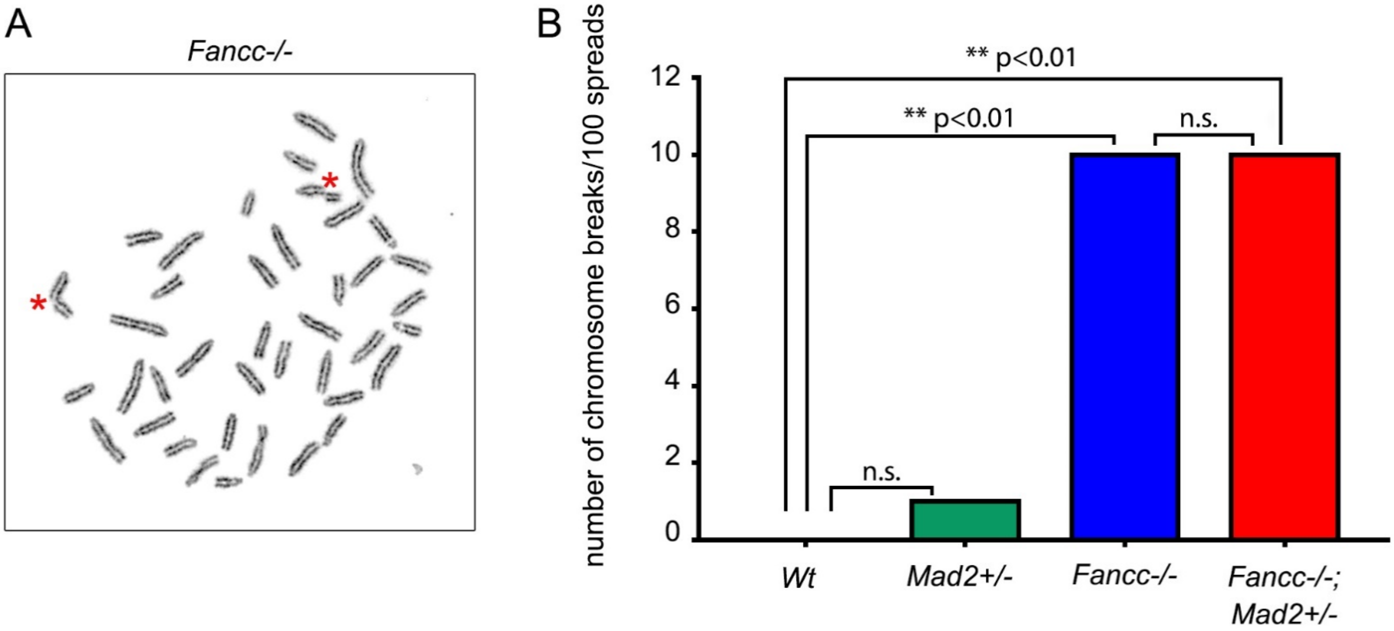

Supplementary Figure 7. Spontaneous chromosome breakage does not differ between *Fancc-/-* and *Fancc-/-; Mad2+/-* cells. Example metaphase spread with fractured chromosomes (asterisks) is shown in **A**. Metaphase spreads (100 spreads/genotype) were Giemsa-stained and numbers of breaks/100 spreads were compared via Fisher's exact test (**B**). ns, non-significant; ** = p<0.01.

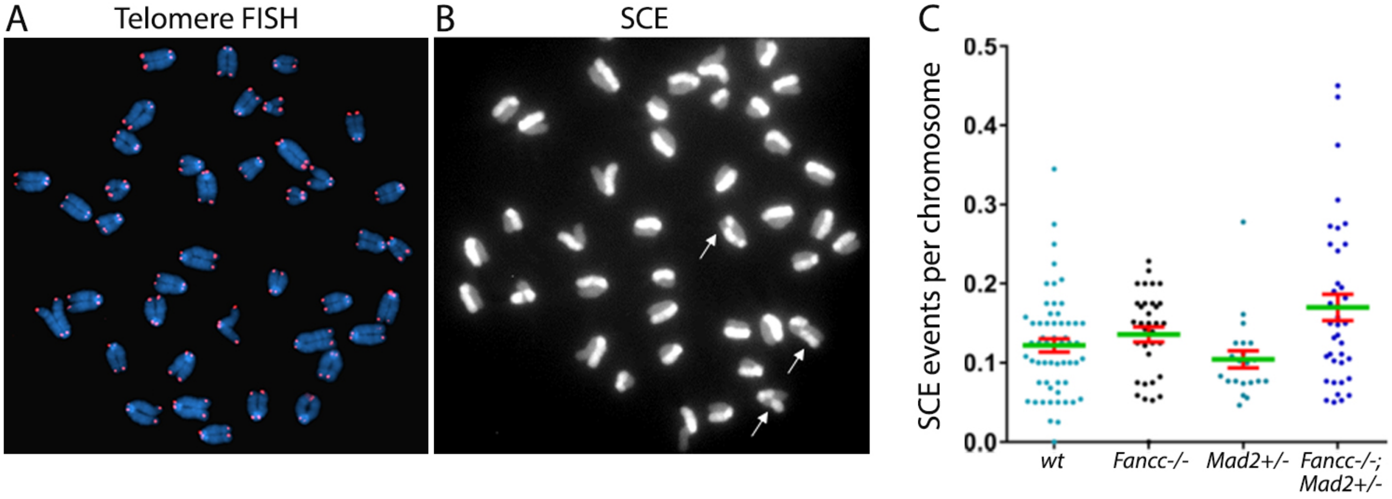

Supplementary Figure 8. *Fancc-/-; Mad2+/-* hematopoietic cells do not have grossly abnormal telomere maintenance or sister chromatid exchange. A representative example of mouse bone marrow telomere FISH is shown in (**A**). Telomere fluorescence signals are red and DNA stain (DAPI) is blue. A representative sister-chromatid exchange (SCE) assay is shown in (**B**). Arrows point to SCE events. (**C**) The frequency of SCE events does not differ between *wt*, *Fancc-/-*, *Mad2+/-* and *Fancc-/-; Mad2+/-* hematopoietic cells. The results were obtained from at least 30 metaphases for each analysis. In each set, data were pooled from at least five mice. All comparisons were non-significant by one-way ANOVA with Tukey's post hoc correction.

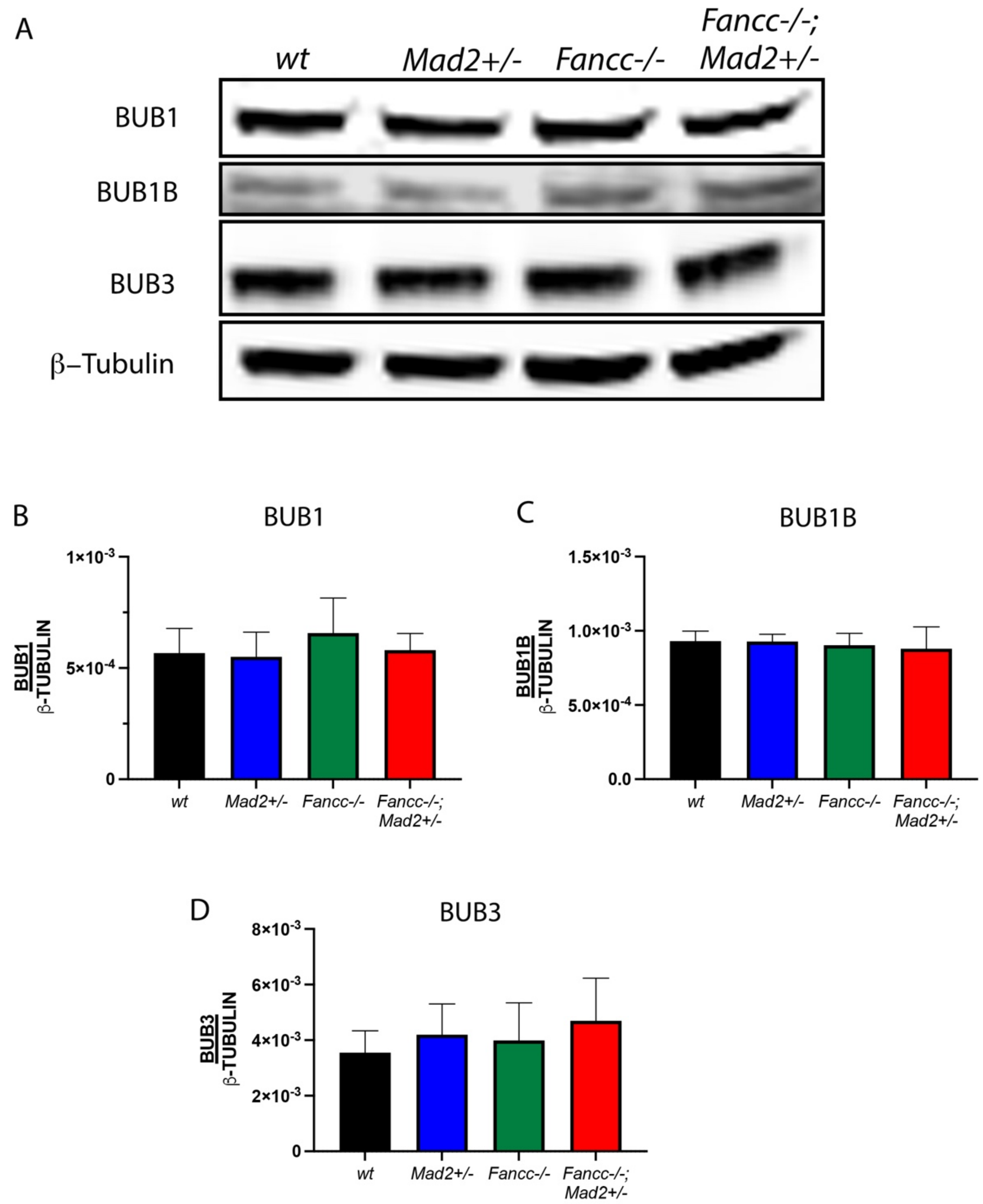

Supplementary Figure 9. *Fancc-/-; Mad2+/-* does not affect the expression of spindle assembly checkpoint proteins BUB1, BUB1B or BUB3 (A) Western blot representative of BUB1, BUB1B and BUB3 expression with β-tubulin serving as a loading control. (B) Quantification of indicated proteins in each genotype relative to *wt*. Graphs represent mean protein expression. BUB1B: n=2 from a single experiment. BUB1 and BUB3: n=3 pooled from two independent experiments. Error bars represent standard error of the mean. Statistical analysis of BUB1 and BUB3 by one-way ANOVA with Tukey's post hoc correction, yielded no significant difference between any of the genotypes.
